# Soil, competition, and niche shifts shape the floral mosaic of an annual plant diversity hotspot

**DOI:** 10.1002/ajb2.70171

**Published:** 2026-03-05

**Authors:** Emma R. Fryer, Ryan O'Dell, Dena L. Grossenbacher, Alyssa Shon, Skyler McKinnon, Nishanta Rajakaruna

**Affiliations:** ^1^ Institute of Integrative Biology, ETH Zurich Zurich 8092 Switzerland; ^2^ Biological Sciences Department California Polytechnic State University, San Luis Obispo San Luis Obispo 93407 CA USA; ^3^ Bureau of Land Management, Central Coast Field Office Marina 93933 CA USA; ^4^ Natural Resources Management and Environmental Sciences Department California Polytechnic State University San Luis Obispo 93407 CA USA; ^5^ SWCA Environmental Consultants San Luis Obispo 93401 CA USA; ^6^ Unit for Environmental Sciences and Management North‐West University Potchefstroom 2531 South Africa

**Keywords:** community assembly, competition, edaphic, endemism, fundamental niche, geoecology, heterogeneity, niche, niche shifts, realized niche

## Abstract

**Premise:**

Plant species with affinity for harsh substrates often have well‐defined edaphic (soil) niches and are ideal for exploring questions of community assembly. Vertic clay soils are chemically and physically challenging to plant establishment and productivity, and annual plant communities associated with these soils of the San Joaquin Desert (California, USA) form a distinctive mosaic pattern of species that reflects differences in soil properties across the landscape.

**Methods:**

We analyzed soil properties to determine how heterogeneous soils at two field sites in the San Joaquin Desert differed among the realized niches of 12 native annual forb species with an affinity for vertic clay soils. We then conducted a pot study with the same species to test if species differed in their realized and fundamental edaphic niches, and to examine the competition effects of an invasive annual grass (*Bromus rubens*) on these species’ edaphic niches.

**Results:**

From our field study, we found some differences in the vertic clay soils between the realized niches of species at both sites. In our pot study, we found species had similar fundamental edaphic niche optima in our treatment soils and that several species’ competitive ability varied with the edaphic stress in our treatment soils. For some species, differences in competitive ability led to shifts in edaphic niche optima, likely contributing to more divergent realized niches.

**Conclusions:**

The combination of competitive pressure and abiotic stress drove differences between the realized niche and fundamental niche for species in a novel, heterogeneous study system.

Soil heterogeneity and differences in niche optima among plant species may be an important driver in structuring plant communities and patterns of occurrence across the landscape (Xue et al., 2019, 2021). Edaphic (soil) heterogeneity influences plant communities’ species composition, richness, and diversity across scales (Harrison and Inouye, 2002; Harrison et al., 2003; Fine et al., 2005; Conti et al., 2017). Abiotic factors like soil drive plant community assembly outcomes through abiotic filtering (Kraft et al., 2015) and by influencing the strength and direction of biotic interactions (Callaway et al., 2002), which can lead to moderation of invasibility and competitive outcomes (Borges et al., 2019). Abiotic heterogeneity also creates more potential niches and therefore increased potential for coexistence through greater opportunities for niche separation (Amaresekare, 2003; Harrison et al., 2006; Chase and Myers 2011; Stein et al., 2014; Stein, 2015), which can also reduce systems’ invasibility (Davies et al., 2005; Mouquet et al., 2006; Melbourne et al., 2007). Much work on heterogeneous serpentine systems has shown that differences in soil properties affect patterns of plant cover and species distribution (Rajakaruna and Bohm, 1999; Lazarus et al., 2011; Yost et al., 2012) and reinforce the effects of edaphic heterogeneity on species distributions (Lazarus et al., 2011; Rossington et al., 2018).

Yet, while stressful soils are especially influential on plant communities (Hulshof and Spasojevic, 2020) and harsh substrate ecosystems have provided much insight into evolution and speciation (Baldwin, 2005; Brady et al., 2005; Burge and Manos, 2011; Harrison and Rajakaruna, 2011; Rajakaruna et al., 2014; Sianta and Kay, 2019, 2021), the role of soil properties in the assembly of plant communities is a relatively unexplored topic (Hulshof and Spasojevic, 2020). Most work on this subject has contrasted communities on and off harsh substrates (Harrison, 1997; Harrison et al., 2003; Anacker, 2011; Schechter and Bruns, 2012; Luzuriaga et al., 2015). In plant communities on another harsh substrate (gypsum soils), plant–plant competitive interactions were more influential on community composition as water stress increased (Luzuriaga et al., 2012). Going et al. (2009) and Reynolds et al. (1997) examined interspecific competition and resource limitations in edaphically heterogeneous serpentine systems and found that species outcompeted one another based on environmental tolerances of soil resource availability. However, we are not aware of any work addressing the combined effects of competition and edaphic stress within heterogeneous systems that explicitly addresses stress‐inducing edaphic factors (e.g., Na content) rather than resource limitations (e.g., low N content).

Under contemporary niche theory, the coexistence of two species is possible if fitness differences are low, niche differences are high, or both are true: With greater differences in niche, barring large fitness differences, two species are more likely to be able to coexist in a community (Snyder and Chesson, 2004; Kraft et al., 2015; Adler et al., 2018). Species with minimal fitness differences stably coexist within the same system by segregating into different niches, provided at least one of the species has a fundamental niche (the niche space a species may occupy in the absence of biotic factors like competition) that allows it to shift along one or more niche axes. Such flexibility maintains differences in realized niche: that portion of the fundamental niche which a species can occupy in the presence of competition and other biotic factors (Silvertown, 2004; Letten et al., 2017). However, when environmental stressors such as high‐stress soils (e.g., ultramafic soils, saline soils) are present, the outcome of two species in competition within a niche is predicted to depend on the degree of stress present and the relative tolerance of each species for that stressor (in essence, their fitness), assuming neither species has an impact on the stressor (Chase and Leibold, 2003). In such a scenario, the more stress‐tolerant species is projected to eventually outcompete the less stress‐tolerant species. If additional niches are available due to local abiotic heterogeneity, it could allow for coexistence of the two species in separate niches.

Community assembly in heterogeneous, high‐stress systems might feasibly entail both of the above mechanisms. These lower‐level mechanisms of assembly (Vellend, 2016) have conservation implications for edaphically heterogeneous communities: More than 68% of the planet's biodiversity hotspots (per Myers et al., 2000 and Mittermeier 1999, 2004) occur on stressful or otherwise “special” soils (Damschen et al., 2011). High rates of endemism, restricted distributions due to the patchy nature of stressful substrates (Kay et al., 2011), and the advance of climate change make edaphic specialists a notable conservation concern (Damschen et al., 2012). Historically, edaphically heterogeneous systems have served as microrefugia for species (Raven and Axelrod, 1978; Pepper et al., 2013; Corlett and Tomlinson, 2020), but whether extant edaphic specialists will find such microrefugia as climate change advances may depend on the breadth of their fundamental niche relative to their realized niche. Plant species associated with stressful soils are frequently specialists with adaptations to tolerate edaphic stress. Edaphic specialization, combined with a strong body of evidence that such adaptation often incurs costs (i.e., a reduction in competitive fitness; Kruckeberg, 1954; Anacker, 2014), implies that edaphic specialists should have a narrower fundamental niche. That restriction might be catastrophic with a shift in climate, if no similar niche falls within the dispersal limitations of species. However, experimentation has shown that some edaphic specialist species can succeed on lower‐stress soils when competition is absent, which suggests the opposite: that some edaphically restricted species may have comparatively broad fundamental niches (Proctor and Woodell, 1975; Jurjavcic et al., 2002). A relatively broad fundamental niche could increase the possibility of persistence (and coexistence) through niche separation for edaphic specialists. A species with a narrow realized niche yet a larger fundamental niche is more likely to be able to occupy some new niche within its dispersal range (and its fundamental niche) upon the loss of a prior niche.

We sought to explore these lower‐level elements of community assembly within a suite of annual forb species in the San Joaquin Desert (California, USA) for several reasons. The species we selected for this study have a distinct mosaic pattern of distribution of near‐totally monospecific patches (Figure 1) that appear to reflect edaphic heterogeneity. Because distribution patterns reflect the realized niche of species (Pulliam, 2000), we interpreted these patches as evidence of a narrow realized niche. We suspected edaphic properties defined these niches: The vertic clay soils these species occur on are visibly heterogeneous and have stress‐inducing properties (e.g., high cation exchange capacity [CEC], high Na, high clay content). We also had evidence of edaphic specialization by our study species to these stressful soils (Fryer et al., 2025). This system is noteworthy because the vertic clay soils at these sites are atypical and highly stressful to plants yet understudied in plant ecology.

**Figure 1:**
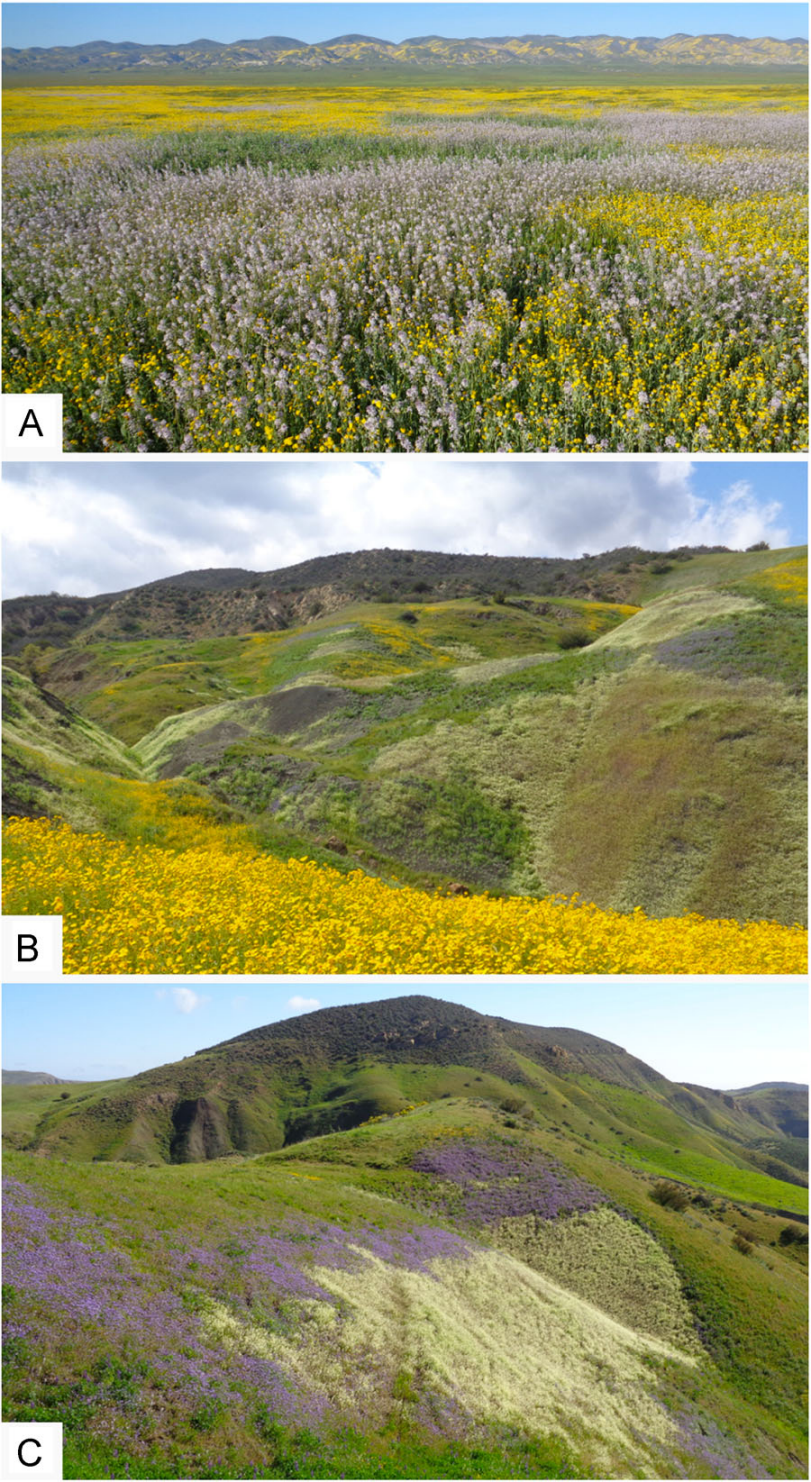
Study sites showing mosaic pattern of blooms after a high‐precipitation winter. (A) Carrizo Plain (San Luis Obispo County, California). Species shown include *Caulanthus anceps* (pink), *Leptosyne calliopsidea* (yellow, in background), and *Monolopia stricta* (yellow, in foreground). (B) Cantua Creek (Fresno County, California). Species shown include *Lepidium jaredii* subsp. *album* (pale yellow‐green), *Monolopia major* and *Madia radiata* (both yellow), and *Phacelia ciliata* (purple). (C) Cantua Creek. Species shown are *Lepidium jaredii* subsp. *album* (pale yellow‐green) and *Phacelia ciliata* (purple).

Lastly, we wanted to explore how competition and our sites’ heterogeneous vertic clay soils might have shaped this community's assembly in the context of the system's invasion by species like *Bromus rubens* L. While the plant communities on vertic clay soils within the San Joaquin Desert contain a relatively high percentage of native, rare, and endemic annual forbs (Thorne et al., 2009; Kraft et al., 2010; Baldwin et al., 2017), non‐native annual grasses like *B. rubens* dominate the landscape for much of the region (Salo, 2005; Buck‐Diaz et al., 2011). We were therefore interested in the invasibility of this system, both in the context of a novel and harsh substrate—vertic clay soils—and the edaphic heterogeneity that we hypothesized was present. Given evidence from heterogeneous serpentine systems that edaphic variation and the competitive strength of specialists on harsh soils can enhance community resistance to invasion (Going et al., 2009), we suspected this system's heterogeneity of stressful vertic clay soils might contribute to the persistence of native species despite invasion by *B. rubens*. For this reason, we focused on competition from *B. rubens* in our study.

We examined 12 native annual forb species associated with vertic clay soils in this study using two components: an observational component in which we sampled soils, and an experimental pot study in which we applied competition and soil treatments. To explore whether these species had realized niches defined by edaphic properties, we sampled soils from 5–10 patches per species at Cantua Creek (Fresno County, California, USA) and at Carrizo Plain (San Luis Obispo County, California, USA). We then analyzed the soil samples for physical and chemical properties to characterize the edaphic realized niche along the axes of those soil properties. If differences in edaphic properties were the primary determinant of the realized edaphic niches of our study species, we anticipated finding significant differences among species for the soils sampled from species patches.

We then used the dominant species of the patches from which those soils were sampled in a pot study to experimentally test for (1) differences among species in fundamental edaphic niche within the range of edaphic factors captured in our pot study soils, (2) edaphic niche shifts when competition from *B. rubens* was present, and (3) species‐level differences in the effect of competition over soil treatments. We grew 12 species on three different field‐collected vertic clay soils that captured a chemical stress gradient of several soil properties that varied across our field sites to determine whether these species differ in their fundamental edaphic niche optima using biomass to measure performance. We hypothesized that these species would differ in their performance when competition was absent over the three soils (i.e., we would observe differences in fundamental edaphic niche optima among species). To test whether competition from *B. rubens* led to niche shifts, we seeded a duplicate set of replicates in the pot study with *B. rubens* in addition to the study species, across all treatment soils. We hypothesized that competition from *B. rubens* would reduce performance of our study species, but that its effect would vary by soil treatment, resulting in niche shifts as edaphic stress modifies competition. We further anticipated species‐level differences therein because species likely differ in their specific edaphic specializations and would therefore also differ in how biotic–abiotic interactions influence their performance.

## MATERIALS AND METHODS

### Species selected

Twelve native annual plant species at our field sites were selected based on affinity for vertic clay soils (Table 1; Consortium of California Herbaria, 2022; Jepson Flora Project, 2022; Fryer et al., 2025), presence at one or both of our field sites, and presence as the dominant species patches making up the mosaic pattern of species observed at both sites.

**Table 1:** Species, family, and sites from which each species was sampled. CC, Cantua Creek; CP, Carrizo Plain.

Species	Family	Field site (s) sampled
*Benitoa occidentalis* (H.M. Hall) D.D. Keck	Asteraceae	CC
*Caulanthus anceps* Payson	Brassicaceae	CP
*Deinandra halliana* (D.D. Keck) B.G. Baldwin	Asteraceae	CC
*Extriplex* “succulenta” sp. nov. O'Dell	Chenopodiaceae	CC
*Layia munzii* D.D. Keck	Asteraceae	CP
*Lepidium jaredii* Brandegee subsp. *album* Hoover	Brassicaceae	CC
*Lepidium jaredii* Brandegee subsp. *jaredii*	Brassicaceae	CP
*Leptosyne calliopsidea* (DC.) A. Gray	Asteraceae	CP
*Madia radiata* Kellogg	Asteraceae	CC
*Monolopia major* DC.	Asteraceae	CC
*Monolopia stricta* Crum	Asteraceae	CP
*Phacelia ciliata* Benth.	Hydrophyllaceae	CP, CC

### Field sites

We focused our study on a plant community found on vertic clay soils in the San Joaquin Desert (California, USA; Germano et al., 2011). We selected two areas that support native annual plant communities associated with vertic clay soils: Cantua Creek (Fresno County, California, USA) and Carrizo Plain (San Luis Obispo County, California, USA). These sites support a diverse community of native annual forbs, which are closely associated with vertic clay soils, including seven taxa in this study that are apparent strict endemics to vertic clay soils (California Native Plant Society, 2025; Consortium of California Herbaria, 2022; Jepson Flora Project, 2022; Fryer et al., 2025).

The soils at Cantua Creek and Carrizo Plain are derived from marine sedimentary rock, with high smectite clay content and high Na content (Reid et al., 1993; Ludington et al., 2005). Both sites have visibly heterogeneous soils and exhibit dramatic and distinctive mosaic‐like floral displays following wet winters, when native species associated with vertic clay soils bloom in distinct patches of nearly monospecific stands of flowers. Both sites host a high proportion of endemic species relative to the total number of native plant species (Thorne et al., 2009; Kraft et al., 2010; Baldwin et al., 2017) and a high number of rare, threatened, and endangered plant species (California Department of Fish and Wildlife, 2023; California Native Plant Society, 2025).

Cantua Creek is located in western Fresno County in the Inner South Coast Ranges on the western edge of the San Joaquin Desert. The Köppen‐Geiger climate classification is Csa (hot‐summer Mediterranean climate) to BSk (cold semi‐arid climate; Beck et al., 2023). The vertic clay soils of Cantua Creek occur on moderate to steep slopes of hills and are derived from marine clay shale (Dibblee and Minch, 2007). The soil orders present include Mollisols, Alfisols, and Entisols (Vinson, 2003). Representative vertic clay soil series of Cantua Creek include Roacha clay and Lilten clay at higher elevations and Belgarra clay, Morenogulch clay, and Gewter clay at lower elevations (Arroues, 2006).

Carrizo Plain is in northeastern San Luis Obispo County. Like Cantua Creek, Carrizo Plain is also located in the Inner South Coast Ranges on the western edge of the San Joaquin Desert. The Köppen‐Geiger climate of Carrizo Plain is BSk (cold semi‐arid climate; Beck et al., 2023). The vertic clay soils on the valley floor of Carrizo Plain are derived from alluvium eroded from marine sedimentary rocks of the surrounding Temblor and Caliente Ranges (Eghbal et al., 1989; Reid et al., 1993, 1996). Fine clay particles and dissolved salts were deposited on the gently sloped valley floor around Soda Lake (Hackel et al., 1962). The representative soil series is Chicote clay, a smectitic Alfisol (Soil Survey Staff, 2003; Vinson, 2003). Although the soils of Carrizo Plain are derived from saline clay alluvial deposits and those of Cantua Creek are derived from saline marine clay shale, both sites have similarly high smectite clay content and high Na concentrations due to similarities in parent material and soil formation processes in the arid climate of the San Joaquin Desert (Vinson, 2003; Arroues, 2006).

#### Vertic clay soils

Vertic clay soils are defined by high clay content (>50%), of which a high percentage (≥20%) is smectite clay (Shirsath et al., 2000). Smectite clay is a physically and chemically extreme substrate for plants (Ahmad, 1975; Eswaran and Cook, 1988; Velde and Barré, 2010). Relative to kaolinite, smectite clay can absorb larger amounts of water, resulting in expansion upon hydration and shrinking upon drying, creating deep cracks, which translates to physical stress for plants (tearing roots) and introduces soil pore discontinuity that may cause additional water stress (Whitmore and Whalley, 2009; Velde and Barré, 2010; Soil Survey Staff, 2022). High clay content in soil also results in lower matric potential than in coarser‐textured soils. As soil particle diameter decreases, soil matric potential decreases, resulting in lower soil water potential that may exceed the lower water potential limit for plant roots and prevent plants from taking up water held by micropore spaces (Kirkham, 2004; Rajakaruna and Boyd, 2008; Velde and Barré, 2010).

Vertic clay soils may also have extreme chemical properties, particularly in semi‐arid to arid climates. In California's San Joaquin Desert, marine‐derived shale has a high clay content and high Na concentration (sodicity), which vertic clays soils inherit (Coulombe et al., 1996; Alexander et al., 2006). Additional Na accumulates in geologic basins where runoff containing dissolved salts evaporates and the salts accumulate over time (Eghbal et al., 1989; Reid et al., 1993, 1996). Combined, these physical and chemical properties of vertic clay soils may exert strong selective pressures on plants.

### Sampling soils in species patches

Field‐collected soil samples from the species patches for each study species (hereafter referred to as their “home soils”) were collected from sites at Cantua Creek, Carrizo Plain, or both, based on species distribution and verifiability of species patches during field visits. For each species, we sampled approximately 0.5 L of rhizosphere soil from a maximum depth of 25 cm within the centroid of a species patch dominated by that species. Replicate sampling (n ≥ 5) for a single species was done at a minimum distance of 15 m and efforts made to ensure patches sampled for a single species were separated by one or more patches dominated by other species to avoid pseudo‐replication. All soil samples were air‐dried and sieved to 2 mm prior to analysis.

### Bulk soil for pot study

Three separate soils were selected as representative of the chemical stress gradient present at field sites. The three soil types had high clay content (~50%+) and spanned a gradient of CEC and N, P, K, Ca, and Mg content in parallel with the gradient of relative sodicity among the three soils. Coefficient of linear extension (COLE), pH, and clay content did not follow the same gradient, though Na:K and Ca:Mg did. Treatment soil types are hereafter referred to by relative sodicity: non‐sodic (NS), somewhat sodic (SS), or sodic (S). All three soils were collected from locations near our Cantua Creek site alone because the conditions of our research permit for Carrizo Plain did not allow soil collections over 0.5 L in volume. Rhizosphere soils were collected from the 0–25‐cm depth interval and sieved on site to <6 mm. The sieving mesh size of 6 mm was selected as a balance between removing debris and avoiding destruction of soil aggregates. Approximately 0.5 L of homogenized sample from each soil type was sieved to 2 mm and retained for analysis.

### Soil analyses

Soil samples collected from field sites and soils used for the pot study were analyzed by A&L Western Laboratories (Modesto, CA, USA) for saturated paste pH (Rhoades and Miyamoto, 1990), KCl (2 M) extractable NO_3_^‐^ (Keeney and Nelson, 1982), weak Bray P1 extractable HPO_4_^‐2^ (Bray and Kurtz, 1945), and bicarbonate extractable HPO_4_^‐2^ (Olsen and Sommers, 1982), ammonium acetate (1 M, neutral pH), extractable K^+^, Ca^2+^, Mg^2+^, Na^+^ (Thomas, 1982), CEC (calculated as the sum of extracted cations), and soil texture. Phosphorus content values used in data analysis were from either weak Bray P1 or Olson/sodium bicarbonate results, as appropriate for the pH of each soil sample (Olsen et al., 1954; Frank et al., 1998). Molar ratios of Na:K and Ca:Mg were calculated from extractable elemental analysis.

Coefficient of linear extension (COLE) was used as a metric of shrink–swell potential, and measured as described by Schafer and Singer (1976).

### Pot study

Seeds for all study species, except for *Layia munzii*, were provided by the Bureau of Land Management Central Coast Field Office. All seed collections made by the Bureau of Land Management were from a minimum of 100 parent plants in combined collections of a minimum of 1000 seeds. Seeds used from those collections were from parent plants growing at or near one of our field sites (collection site was determined by species distributions). Parent plants for all species were growing on vertic clay soils. For *L. munzii*, we collected approximately 800 total seeds from approximately 50 parent plants in July 2020 at Carrizo Plain.

The pot study was conducted outdoors in the city of Fresno (Fresno County, California, USA) from December 2020 to July 2021. Fresno is located at the far eastern edge of the San Joaquin Desert and has a Köppen climate classification of BSk (cold semi‐arid) to Csa (hot summer Mediterranean; Beck et al., 2023). Pots were set up in full sunlight, on a 1‐m high platform to ensure even and continuous sun exposure throughout daylight hours.

The three soil types used captured a gradient of soil chemical stress present at field sites (Table 3). Clay soils are susceptible to pore collapse when the soil moisture content exceeds the plastic limit (the lowest moisture content at which a soil can undergo deformation without cracking), and passive watering of potted clay soils is often ineffective. For this reason, soils were manually wetted to ensure homogenous wetting throughout the soil for each pot. Each soil type was mixed with water by hand to achieve a consistent, homogeneous level of moisture within a semisolid state without exceeding the plasticity limit. Soils were wetted manually until stable, moist 6–8‐mm crumb aggregates formed. Plastic tree pots (12.7 × 12.7 × 30.5 cm) were filled to the top with the moistened soil. Soil was settled into the pots by lifting pots from the top edges and lightly tapping the bottom of the pot on a solid surface. Additional moistened soil was added to all pots so that the soil surface was within 2 cm of the top edge of the pot. The pots were then placed in plastic subirrigation trays (29.5 × 54.6 × 6.4 cm), which were filled with water to a depth of approximately 5 cm. The potted soils were left for 24 h to rest and allow the soil to further moisten to field capacity by capillary action. Due to low seed germination in germination trials, the pots were heavily seeded (50–100 seeds; seedlings were thinned to one per pot following germination) for all study species in December 2020. Seeds were left uncovered by soil and misted from above daily for 4 weeks in addition to the subirrigation.

### Randomization and blocking

**Figure 2:**
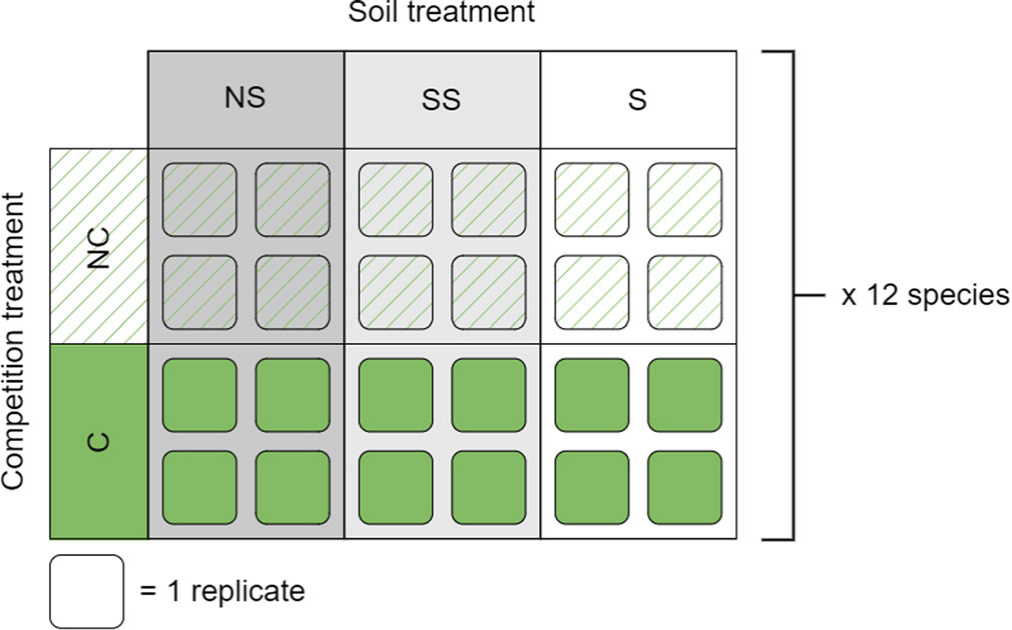
Treatment combinations applied to each species in pot study. C, competition present; NC, no competition; NS, non‐sodic soil; SS, somewhat sodic; S, sodic.

Each of the 12 focal species was seeded in four replicate pots for every combination of soil treatment and competition treatment for *N* = 24 pots per species total (Figure 2). Pots were separated following seedling thinning into four blocks of replicates (one replicate of each species × soil treatment × competition treatment per block) and pots within each block were randomized in placement within the block. So that dissolved minerals from one soil type could not leach into the subirrigation water of the tray and affect another soil type, each block was internally randomized into sub‐blocks of one soil type within a tray.

Before seeding in pots, all seeds were cold‐stratified at 3°C for 3 months. For species with low germination rates in the germination trials (*Benitoa occidentalis*, *Caulanthus anceps*, *Deinandra halliana*, *Layia munzii*, *Monolopia major*, *M. stricta*, and *Phacelia ciliata*), backups were seeded in potting soil in seedling trays (3.8 × 3.8 cm cells) on the same date all pots were seeded. Transplants for one or more pots were necessary for *B. occidentalis*, *C. anceps*, *D. halliana*, *L. munzii*, *M. major*, *M. stricta*, and *P. ciliata* and were transplanted within 5 weeks of seeding in all cases. Seedlings were thinned to one individual per pot. Each pot with the competition treatment was thinned to three individual *B. rubens* seedlings surrounding one focal species seedling.

Overhead watering was conducted to keep the soil surface moist through seed germination and establishment. After seedling establishment, overhead watering was reduced and applied as needed for all pots (watered in a uniform manner to all pots when applied). All overhead watering was tapered off after 3 months (late February 2021), and watering was limited to subirrigation for the remainder of the study. Beginning in May 2021, the refilling frequency of subirrigation water was gradually reduced to reflect field conditions. Beginning in June 2021, the volume of water used to refill subirrigation trays was also reduced. By July 2021, all plants had senesced, shortly before subirrigation tray refills were scheduled to be halted.

### Biomass data collection

Biomass of each plant was weighed following senescence of each plant. Senescence was defined as having occurred when either the last flower had bloomed and withered, with no developing buds remaining, or over 50% of leaves on the plant had browned over at least one third of their surface area, regardless of fruit development on the last flowers. To reduce loss of reproductive parts that might alter final biomass, flowers were bagged with cinchable polyester organza exclusion bags enclosing the flower and top of the pedicel after petals began to wither or after the innermost disk flowers had been open for a minimum of 24 h. For Asteraceae, bags enclosed the capitulum and top of the peduncle. If a plant had not yet met our definition of senescence but had mature fruits, the fruits and flower parts captured in the bag were removed and dried early to prevent loss of that biomass.

For harvesting, stems were cut at the soil surface, shoots were collected, then the soil and roots were immediately removed from pots to clean the roots using a method modified from Rechel (1993). The soil and roots were submerged in a dilute (≤0.25%) HCl solution for 15–45 min to loosen the soil by dissolution of carbonates, in combination with sonication at 70 kHz for 15‐min intervals that were repeated as needed to loosen the clay soil particles from the roots. The soil and roots were further sonicated and rinsed with water with manual agitation of the root mass over a 2‐mm sieve. Controlled agitation and rinsing with water over the sieve were repeated as necessary to prevent roots from tearing or any substantial loss of root mass, until the roots were entirely free of soil particles. Roots of competition‐treated plants were painstakingly and carefully separated from *B. rubens* roots by hand. All collected and cleaned plant materials (reproductive parts, shoots, and roots) were dried to constant mass at 41°C for 24–48 h, weighed separately, and the combined total biomass of all three was recorded.

### Data analyses

#### Soils

R in RStudio (version 4.5.2, R Core Team, 2025; version 2025.05, RStudio Team, 2025) was used for all analyses. Results for pH, N, P, K, Ca, Mg, Na, CEC, clay (%), and COLE of field‐collected soils were standardized (transformed to have a mean of 0 and a standard deviation of 1) with the scale function from base R (R Core Team, 2025) and used as variables in a weighted PCA of all soil samples using the Wpca function from R package WCluster (version 1.3.0; Dey et al., 2025). Soil samples for each species were weighted based on sample size to offset differences in sample size among species, and the Wpca function was used for eigenvalues‐based weighting (Dey et al., 2025). To determine whether the soils of the species patches differed in the soil properties tested (i.e., realized edaphic niche), we used a Kruskal–Wallis rank sum test on the coordinates of the soil samples of each species on the first three principal components (in three separate tests) using the function kruskal.test from the base package stats (R Core Team, 2025). To determine whether soils sampled from species patches differed in the breadth of the soil's properties (i.e., edaphic niche breadth), we used Levene's test on the coordinates of soil samples on the first three principal components (in three separate tests) using the function leveneTest from the R package car (version 3.1‐3; Fox and Weisberg, 2019).

To explore whether differences in edaphic niche and niche breadth were the result of locality effects, we then conducted PCAs of soil properties with soil samples from each site separately. We again tested for differences in the mean coordinates of soil samples for each species on the first three principal components (realized edaphic niche) with a Kruskal–Wallis rank sum test and with Levene's test on the same values to test for differences in variance (niche breadth).

#### Biomass

To experimentally test for fundamental edaphic niche optima, niche shifts, and differences in both niche shifts and realized edaphic niche optima by species, we fitted a linear mixed‐effects model with restricted maximum likelihood, with species, soil treatment, competition treatment, and all two‐ and three‐way interactions of the three as fixed effects. We included block as both a fixed effect and as a random effect with soil nested in block. We log‐transformed biomass as the response variable to [log(y + 1)] using the function log1p in base R (R Core Team, 2025). Our final model was log(Total biomass + 1) *~*Species ∗ Competition ∗ Soil + Block + (1 | Block:Soil). We fit the model using the function lmer in R package lme4 (version 1.1‐37; Bates et al., 2015). We then conducted a two‐way ANOVA with the function anova in the base R package stats (R Core Team, 2025). Although we explored reproductive biomass and both above‐ and below‐ground biomass as response variables, we found that no new patterns emerged relative to total biomass; we therefore focused our analysis on total biomass.

Based on the three‐way interaction of species, soil, and competition in the model, and on the differences in home soils on the first three principal components of field‐collected soils, we then conducted post‐hoc contrasts of the estimated marginal means by competition treatment across soil treatments for the log‐transformed biomass of each species. We also contrasted the estimated marginal means by soil treatment across competition treatments. To find differences in estimated marginal means and conduct post hoc contrasts, we used the R package emmeans (version 2.0.1; Lenth, 2025). Contrasts were conducted using Tukey's honest significant difference method: Contrasts by soil treatment across competition treatment were conducted with *P* adjusted for comparing a family of three estimates.

## RESULTS

### PCA of soil properties

**Figure 3:**
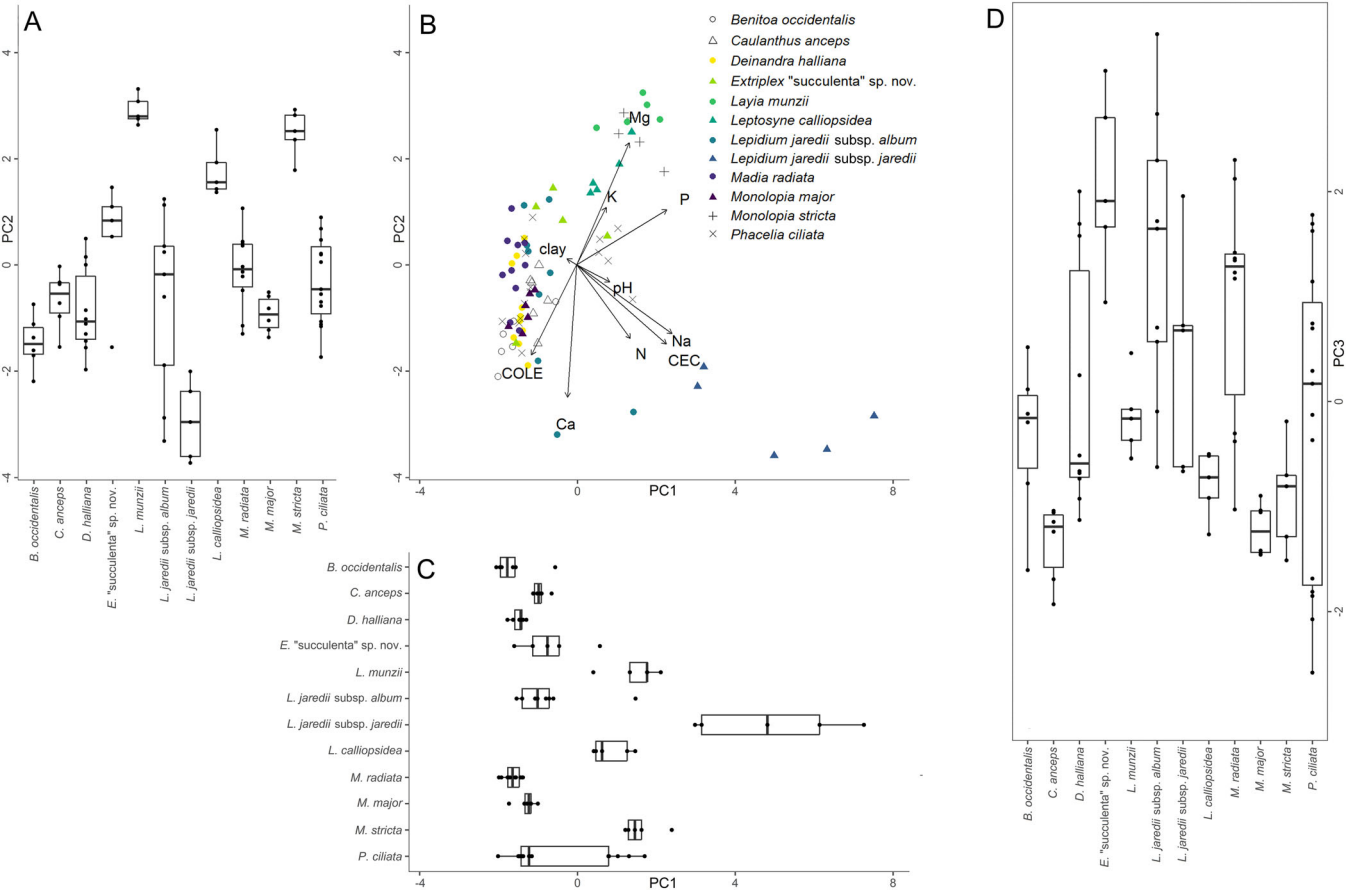
Results of PCA for soil samples collected from focal species patches at both sites. (A) Box plot of soil sample coordinates on the second principal component, by species. PC2 explains 24.0% of variation. PC2 is shown at the same scale in A and B; loadings shown in B apply to A. (B) Biplot of soil samples by species shown over the first two principal components, with loading arrows for soil properties assessed. (C) Box plot of soil sample coordinates on the first principal component, by species. PC1 explains 32.2% of variation. PC1 is shown at the same scale in B and C; loadings shown for B apply to C. (D) Box plot of soil sample coordinates on the third principal component, by species. PC3 explains 18.6% of variation; values higher on PC3 correspond to soils higher in clay content, but lower pH and K (see Table 2 for loading values; for biplots of soil samples collected from species patches plotted over PC2 and PC3, and over PC1 and PC3, see Appendix S1).

In our PCA considering the home soils of species from both sites together, the first three principal components of soil properties together explained 74.8% of the total variation among samples (Figure 3, Table 2). We found that significant (*α* = 0.05) differences in the realized edaphic niche of species (in terms of the niche axes for the soil properties addressed here) were present for all three of the principal components examined and that differences in niche breadth were also present: We found a significant difference in mean coordinates for species on all three principal components (Figure 3; Kruskal–Wallis test, PC1: *χ* ^2^ = 61.960, df = 11, *P* < 0.001, PC2: *χ* ^2^ = 55.546, df = 11, *P* < 0.001, PC3: *χ* ^2^ = 42.130, df = 11, P < 0.001). We also found that species’ home soils had differences in variance over all three principal components (Levene's test, PC1: *F* = 3.156, *P* < 0.001, PC2: *F* = 2.168, *P* = 0.025, PC3: *F* = 2.119, *P* = 0.028). The species at the two sites had some overlap on all principal components, though separated more over the first principal component than the other two considered here. Differences in niche space and niche breadth on the first principal component (Figure 3; 32.2% of variation) were driven by differences in P, Na, and CEC. Differences on the second principal component (Figure 3; 24.0% of variation) were driven by Ca, Mg, and COLE. Differences in niche and niche breadth on the third principal component (Figure 3; 18.6% of variation) were defined most by pH, K, and clay content. The separation of the species at the two sites over the first principal component was driven by differences in P, Na, and CEC in soils at the two sites: The differences between sites in Na content were likely due in part to soils collected from patches of *Lepidium jaredii* subsp. *jaredii*, which had substantially higher Na content in its soils than any other species. Soils for other species also had high Na content relative to lower‐stress soils, but did not approach the levels seen in the soils for *L. jaredii* subsp. *jaredii*. Based on the apparent separation of species by site on the first principal component, we also addressed the home soils of species in separate, site‐specific PCAs to ensure locality effects and site differences were not behind these results.

**Table 2:** Factor loading values for the first three components from PCAs of properties for home soils of species at the two study sites. CEC, cation exchange capacity.

	Species from both sites			Species from Cantua Creek			Species from Carrizo Plain		
Property	PC1	PC2	PC3	PC1	PC2	PC3	PC1	PC2	PC3
pH	0.177	–0.068	–0.591	0.414	–0.125	0.132	–0.317	0.006	0.030
N, ppm	0.481	0.217	–0.105	–0.331	–0.085	0.124	–0.202	–0.485	0.160
P, ppm	0.158	0.227	–0.267	–0.030	–0.401	–0.002	0.146	0.507	–0.268
K, ppm	0.281	0.483	0.250	–0.518	0.128	–0.034	0.234	–0.455	–0.422
Ca, ppm	–0.048	–0.525	–0.219	0.303	–0.483	–0.038	–0.305	0.467	0.114
Mg, ppm	0.509	–0.273	0.173	–0.359	–0.372	0.214	–0.455	–0.209	0.023
Na, ppm	–0.240	–0.358	0.223	0.074	–0.396	–0.615	–0.284	–0.062	–0.262
CEC, meq/100 g	0.287	–0.293	0.053	–0.041	–0.156	0.625	–0.371	0.157	0.052
Clay, %	–0.051	0.023	0.579	–0.357	0.100	–0.386	–0.199	0.067	–0.799
COLE	0.479	–0.315	0.201	–0.307	–0.488	0.031	–0.474	–0.080	–0.022

**Figure 4:**
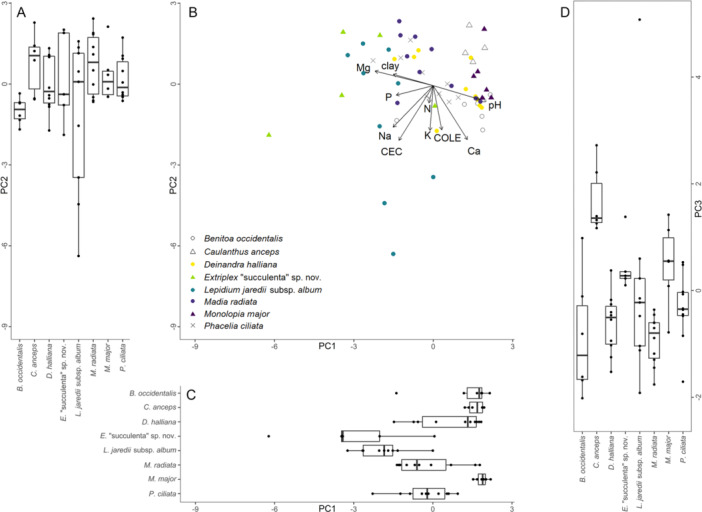
Results of PCA for soil samples collected from focal species patches at Cantua Creek. (A) Box plot of soil sample coordinates on the second principal component, by species. PC2 explains 24.4% of variation. PC2 is shown at the same scale in A and B; loadings shown in B apply to A. (B) Biplot of soil samples by species shown over the first two principal components, with loading arrows for soil properties assessed. (C) Box plot of soil sample coordinates on the first principal component, by species. PC1 explains 33.0% of variation. PC1 is shown at the same scale in B and C; loadings shown for B apply to C. (D) Box plot of soil sample coordinates on the third principal component, by species. PC3 explains 14.8% of variation. Values higher on PC3 correspond to soils higher N, but lower COLE (see Table 2 for loading values; for biplots of soil samples collected from species patches plotted over PC2 and PC3, and over PC1 and PC3, see Appendix S2).

**Figure 5:**
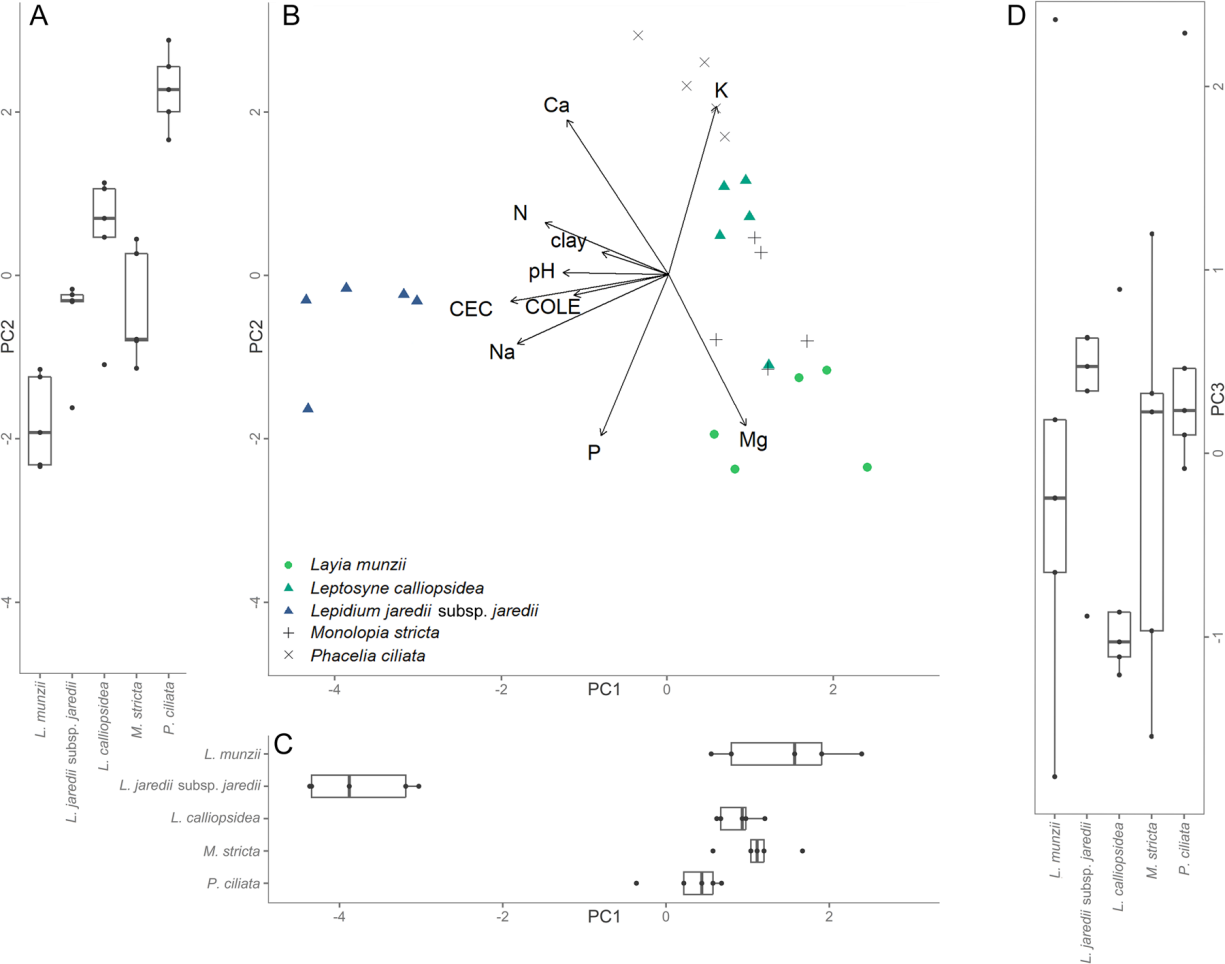
Results of PCA for soil samples collected from focal species patches at Carrizo Plain. (A) Box plot of soil sample coordinates on the second principal component, by species. PC2 explains 20.9% of variation. PC2 is shown at the same scale in A and B; loadings shown in B apply to A. (B) Biplot of soil samples by species shown over the first two principal components, with loading arrows for soil properties assessed. (C) Box plot of soil sample coordinates on the first principal component, by species. PC1 explains 40.5% of variation. PC1 is shown at the same scale in B and C; loadings shown for B apply to C. (D) Box plot of soil sample coordinates on the third principal component, by species. PC3 explains 11.5% of variation; values higher on PC3 correspond to soils lower in Mg and clay content (see Table 2 for loading values; for biplots of soil samples collected from species patches plotted over PC2 and PC3, and over PC1 and PC3, see Appendix S3).

In PCAs of soil properties from both sites conducted separately (Figures 4 and 5), we found that for both sites, within‐site differences among the realized edaphic niche of species were present on two of the first three principal components for both sites, but that niche breadth did not significantly differ as often within sites as it did within the PCAs of both sites.

In our PCA of the Cantua Creek site (Figure 4, Table 2), we found differences in realized edaphic niche were present on two of the three principal components, but that niche breadth varied only on one principal component. In this analysis, we found that the mean coordinates for species differed significantly on PC1 (Kruskal–Wallis test, *χ* ^2^ = 39.580, df = 7, *P* < 0.001) and PC3 (*χ* ^2^ = 29.894, df = 7, *P* < 0.001), but not on PC2 (*χ* ^2^ = 11.113, df = 7, *P* = 0.134), whereas variation was significantly different on PC2 (Levene's test, *F* = 2.726, *P* = 0.017,), but not PC1 (*F* = 1.478, *P* = 0.195) or PC3 (*F* = 1.193, *P* = 0.323). The first three principal components explained 72.2% of variation among samples (Figure 4, Table 2). The differences in realized edaphic niche on the first principal component (33.0% of variation) were driven by Mg and pH, and to a lesser degree by Na and clay content. However, niche breadth did not differ significantly by species in terms of those soil properties. On the second component (24.4% of variation), there was not a significant difference in realized edaphic niche for species (where niche was defined mainly in terms of CEC, Ca, and K); however, species did differ in niche breadth in terms of those properties. On the third component (14.8% of variation), differences in the realized edaphic niche of species were driven by N, COLE, and clay content, but niche breadth did not differ significantly.

In our PCA for Carrizo Plain species (Figure 5, Table 2), we saw differences in realized niche on two of the three principal components, but found no significant differences in the niche breadth. We found that mean coordinates of species differed significantly on PC1 (*χ* ^2^ = 17.250, df = 4, *P* = 0.001) and PC2 (*χ* ^2^ = 19.488, df = 4, *P* < 0.001), but not PC3 (*χ* ^2^ = 4.357, df = 4, *P* = 0.360), and that the variance of soils did not differ significantly by species on any of the components (PC1: *F* = 1.653, *P* = 0.200, PC2: *F* = 0.232, *P* = 0.9173, PC3: *F* = 0.489, *P* = 0.744). Differences in the realized edaphic niche of species on the first principal component (40.5% of variation) were due to Na, CEC, and N. On the second principal component (20.9% of variation), the observed niche differences were defined by K, P, and Ca, and on the third component (11.5% of variation), differences were driven mainly by clay and Mg content.

Values for field soil properties used in all PCAs are in Appendix S4.

### Soil properties for pot study

The three soils in the pot study (Table 3) captured a gradient of N, Ca, Mg, Na, CEC, Ca:Mg, and Na:K. Nitrogen content increased with sodicity, whereas pH, P, clay content, and COLE varied among the soils without forming a similar gradient.

**Table 3:** Properties of treatment soils used in pot study.

Property	Non‐sodic	Somewhat sodic	Sodic
pH	7.4	7.0	7.9
N, ppm	2	6	9
P, ppm	12.1	7.9	8.3
K, ppm	383	493	653
Ca, ppm	4672	4840	5644
Mg, ppm	766	729	149
Na, ppm	18	199	1959
CEC, meq/100 g	30.7	32.3	39.6
Clay, %	61	47	53
COLE	0.198	0.196	0.219

### Soil and competition treatment effects

In the model of log‐transformed biomass (Tables 4 and 5), there was a significant (α = 0.05) three‐way interaction of soil treatment, competition treatment, and species, reflecting differences in how species response to the soil treatments varied by competition treatment. This interaction supports our hypothesis that species differed in how the effect of competition varied over the three soil treatments.

**Table 4:** Pot study results for log‐transformed biomass from the model log(Biomass + 1) ~Species * Soil * Treatment + Block + (1 | Block:Soil).

Factor	Sum of squares	df	F‐value	*P*
Species	83.059	11	147.598	<.001
Soil	3.013	2	29.450	<.001
Competition	39.148	1	765.240	<.001
Block	0.359	3	2.340	0.173
Species * Soil	10.041	22	8.922	<.001
Species * Competition	2.063	11	3.666	<.001
Soil * Competition	2.988	2	29.206	<.001
Species * Soil * Competition	2.294	22	2.038	0.006

**Table 5:** Estimated marginal means (Estimate) using log‐transformed biomass modeled as log(Biomass + 1) ~Species * Soil * Treatment + Block + (1 | Block:Soil) (Table 4), averaged over blocks. df = 189 and confidence level (CL) = 0.95 in all cases. C, competition present; NC, competition absent; NS, non‐sodic soil; SS, somewhat sodic; S, sodic.

Species	Competition	Soil	Estimate	SE	Lower CL	Upper CL
*Benitoa occidentalis*	NC	NS	2.916	0.115	2.689	3.144
		SS	3.213	0.115	2.986	3.441
		S	2.876	0.115	2.648	3.103
	C	NS	2.342	0.115	2.115	2.570
		SS	2.710	0.115	2.482	2.937
		S	2.349	0.115	2.122	2.577
*Caulanthus anceps*	NC	NS	1.533	0.133	1.270	1.796
		SS	1.994	0.115	1.767	2.222
		S	1.462	0.115	1.234	1.689
	C	NS	1.171	0.115	0.944	1.399
		SS	1.369	0.115	1.142	1.597
		S	0.809	0.115	0.581	1.036
*Deinandra halliana*	NC	NS	1.907	0.115	1.679	2.134
		SS	2.681	0.115	2.454	2.909
		S	1.845	0.115	1.618	2.073
	C	NS	0.817	0.115	0.589	1.045
		SS	1.695	0.115	1.468	1.923
		S	0.712	0.115	0.484	0.939
*Extriplex* “succulenta” sp. nov.	NC	NS	1.925	0.115	1.697	2.152
		SS	1.591	0.115	1.363	1.818
		S	1.923	0.115	1.695	2.150
	C	NS	0.981	0.115	0.754	1.209
		SS	0.733	0.115	0.505	0.960
		S	1.214	0.115	0.987	1.442
*Layia munzii*	NC	NS	1.284	0.115	1.057	1.512
		SS	2.123	0.115	1.896	2.351
		S	1.358	0.115	1.131	1.586
	C	NS	0.679	0.115	0.452	0.907
		SS	1.036	0.115	0.809	1.264
		S	0.443	0.115	0.215	0.670
*Lepidium jaredii* subsp. *album*	NC	NS	0.408	0.115	0.180	0.635
		SS	1.563	0.115	1.335	1.790
		S	1.205	0.115	0.977	1.432
	C	NS	0.216	0.115	–0.012	0.443
		SS	0.616	0.115	0.388	0.843
		S	0.609	0.115	0.382	0.837
*Lepidium jaredii* subsp. *jaredii*	NC	NS	0.421	0.115	0.193	0.648
		SS	1.164	0.115	0.937	1.392
		S	0.932	0.115	0.704	1.159
	C	NS	0.269	0.115	0.041	0.497
		SS	0.035	0.133	–0.228	0.298
		S	0.219	0.115	–0.008	0.447
*Leptosyne calliopsidea*	NC	NS	1.057	0.115	0.830	1.285
		SS	1.558	0.115	1.330	1.785
		S	1.652	0.115	1.425	1.880
	C	NS	0.633	0.115	0.405	0.860
		SS	0.010	0.133	–0.253	0.273
		S	0.792	0.133	0.530	1.055
*Madia radiata*	NC	NS	1.625	0.115	1.397	1.852
		SS	2.245	0.115	2.017	2.473
		S	1.764	0.115	1.536	1.991
	C	NS	1.436	0.115	1.208	1.663
		SS	1.472	0.115	1.244	1.699
		S	0.981	0.115	0.754	1.209
*Monolopia major*	NC	NS	1.557	0.115	1.329	1.785
		SS	2.083	0.115	1.855	2.310
		S	1.669	0.115	1.442	1.897
	C	NS	1.141	0.115	0.914	1.369
		SS	1.278	0.115	1.051	1.506
		S	0.699	0.115	0.471	0.926
*Monolopia stricta*	NC	NS	1.076	0.115	0.849	1.304
		SS	1.598	0.115	1.370	1.826
		S	0.978	0.115	0.751	1.206
	C	NS	0.920	0.133	0.657	1.182
		SS	0.561	0.115	0.333	0.788
		S	0.109	0.115	–0.118	0.337
*Phacelia ciliata*	NC	NS	1.446	0.115	1.219	1.674
		SS	1.969	0.133	1.706	2.231
		S	1.475	0.115	1.248	1.703
	C	NS	0.925	0.115	0.697	1.152
		SS	0.699	0.115	0.472	0.927
		S	0.467	0.115	0.240	0.695

**Figure 6:**
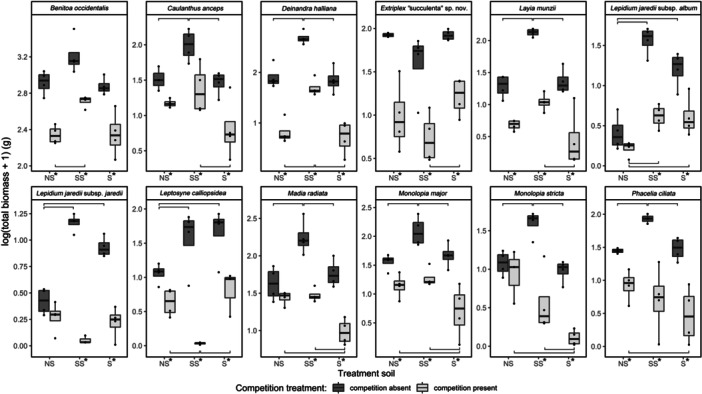
Log‐transformed total biomass by species, soil treatment, and competition treatment; NS, non‐sodic soil; SS, somewhat sodic; S, sodic. Contrasts were conducted with estimated marginal means of log‐transformed biomass modeled as log(Biomass + 1) ~Species * Soil * Treatment + Block + (1 | Block:Soil) (Table 4), averaged over blocks. Brackets indicate differences between soil treatments within competition treatments where *P* < 0.05. Asterisks indicate differences between competition treatments within soil treatments where *P* < 0.05.

In post hoc contrasts of estimated marginal means of the log‐transformed biomass of species between soil treatments (Figure 6; Appendix S5), most species had significant differences in biomass between at least two soil treatments when competition was absent, which supports the existence of some fundamental niche optimum within the treatment soils for those species. Only two species (*B. occidentalis* and *Extriplex* “succulenta" sp. nov.) did not have significantly higher estimated marginal means for biomass on at least one soil treatment relative to another when competition was absent. Nonetheless, even for *B. occidentalis* and *E*. “succulenta”, one or two soils still trended notably higher than at least one other soil (Figure 6). Surprisingly, for all species except *E*. “succulenta”, biomass consistently trended high on the somewhat sodic soil when competition was absent. This pattern suggests species had a common fundamental niche optimum (in terms of treatment soils) on the somewhat sodic soil. These results support our hypothesis that performance would differ by species over the three soils when competition was absent, but do not reflect as many differences in fundamental edaphic niche optima among species as we anticipated.

In post hoc contrasts of biomass between soil treatments when competition was present (Figure 6; Appendix S5), most species had at least one soil treatment with significantly higher biomass than another soil, except for *B. occidentalis* and *L. jaredii* subsp. *jaredii*, though in both cases, notable trends were still apparent. For all species, an apparent realized niche optimum existed or was suggested by trends in biomass among soils (as with *B. occidentalis* and *L. jaredii* subsp. *jaredii*) when competition was present.

In post hoc contrasts between competition treatments (Appendix S6), most species had significant differences in estimated marginal means of log‐transformed biomass between competition treatments, for all soil treatments. For *B. occidentalis*, *C. anceps*, *D. halliana*, *E*. “succulenta”, *L. munzii*, and *M. major*, competition significantly reduced performance on all soils, but did not lead to a niche shift (i.e., the soil treatment with the highest biomass did not change between competition treatments), at least in terms of our pot soils. For *B. occidentalis*, *C. anceps*, *D. halliana*, *L. munzii*, and *M. major*, the somewhat sodic soil remained their edaphic niche optimum (and significantly higher than at least one other soil treatment) when competition was present. For *B. occidentalis*, this difference was reflected in trends of biomass, though not significant. Only *E*. “succulenta” differed, in that its biomass trended similarly high on the non‐sodic and sodic soils, and lower on the somewhat sodic soil regardless of competition, suggesting some shared feature(s) of the non‐sodic and sodic soils defined its niche optimum.

For *L. jaredii* subsp. *jaredii*, *L. jaredii* subsp. *album*, *M. radiata*, and *M. stricta*, competition did not appear to have a significant effect on biomass on the non‐sodic soil, but did on the somewhat sodic and sodic soils: these species had low competitive ability on that soil. However, for all four species, the non‐sodic soil was not the soil on which biomass trended highest if competition was absent. For *L. jaredii* subsp. *album*, competition did not lead to a niche shift: When competition was present, biomass in the somewhat sodic and sodic soil remained significantly higher than in the non‐sodic soil, though the difference between the somewhat sodic and sodic soils was reduced. The other three species all had their fundamental niche optimum on the somewhat sodic soil, but their realized niche optimum varied. *Lepidium jaredii* subsp. *jaredii* had a fundamental niche optimum over the somewhat sodic and sodic soils (though somewhat sodic trended higher), and while soils did not differ significantly when competition was present, trends suggest a possible shift in niche optimum to the sodic soil. *Madia radiata* shifted to a realized niche optimum over the non‐sodic and somewhat sodic soils, and *M. stricta* to the non‐sodic and somewhat sodic soils (though non‐sodic trended higher).

Lastly, two species saw niche shifts in addition to a significant reduction in biomass due to competition on all three treatment soils: *Leptosyne calliopsidea* went from a fundamental niche optimum of the somewhat sodic and sodic soils to a realized niche optimum of the non‐sodic and sodic soils, and *P. ciliata* saw a shift from higher biomass on the somewhat sodic soil to the non‐sodic soil. Our results partially support our hypothesis that competition from *B. rubens* would reduce performance of our study species, but that its effect would vary by soil treatment, resulting in niche shifts. We saw niche shifts in the biomass trends of only half of our study species, which agrees with our model interaction that suggested species differ in the dynamics that determine their realized niche.

## DISCUSSION

We analyzed the physical and chemical properties of the home soils of species and found that, although differences in the vertic clay soils were present among species patches, the degree of overlap was greater than expected given the mosaic patterns of their realized niches. In our pot study, biomass differed significantly for nearly all our study species somewhere over the range of edaphic properties and stress captured in our treatment soils. These results suggest some fundamental niche optima for those species exist within our treatment soils. For most species, that optimum converged on the somewhat sodic soil. Competition from non‐native *B. rubens* significantly reduced performance of nearly all our study species, and in several cases, niche shifts occurred when competition was present (realized niche optima were on different treatment soil than fundamental niche optima). Our combined results suggest that while heterogeneous edaphic properties at our sites do have a role in shaping the realized niche of these species, competition influences their responses to edaphic factors, often leading to niche shifts.

Our analysis of field‐sampled soils in the PCAs partially supported our hypothesis that our study species have edaphically distinct realized niches. Some differences were present in all analyses, but differences in niche breadth in the combined PCA appeared to be largely due to site. As niche breadths differed notably in the PCA with both sites together (Figure 3), it appears likely that site differences were behind the differences in niche breadth we saw in the PCA of both sites (the higher variance of *P. ciliata*'s home soils between the two sites it was collected from was likely also a factor). In our analyses of sites separately, the more homogeneous spread of principal component coordinates by species within sites suggests that the differences in niche breadth seen in the analysis of sites together were due to site differences, with niche breadth within sites being more similar. The significant differences seen on the principal components defined by Mg, pH, N, and COLE for Cantua Creek and by Na, N, CEC, Mg, and clay for Carrizo Plain suggest that soil properties were driving niche differences. However, both sites also had one principal component on which soils did not differ significantly by species: at Cantua Creek on the component defined by Ca, CEC, and K and at Carrizo Plain on the component defined by clay content and Mg. Together, these results suggest that the 12 species differ in their realized edaphic niches, both in niche optimum and niche breadth, but that within sites, species had realized niches with similar breadth. Our PCA results only partially support our hypothesis that the vertic clay soils at our sites are heterogeneous among species patches: Soils were not entirely heterogeneous among species patches, which suggests that other factors influence the realized niches of these species. Because species at both sites consistently create the same mosaic pattern of nearly monospecific patches, factors other than the soil properties we tested likely drove partitioning of species into the distinct realized niches we found them in at both study sites. Despite overlap in home soil properties, the distinct species patches observed may be driven by other soil properties or abiotic factors, or biotic interactions influencing these species, as indicated by our pot study. We likely did not capture all the essential niche axes defining the realized niche of these species, even in terms of soils (though this is a general limitation of working within a niche framework; Angilletta et al., 2019).

Additionally, the soils at both sites are high in properties stressful to plant life (Appendix S4), so some of the overlap in our PCA may be due to shared affinity of species for vertic clay soils and their associated stressful properties. Given the high level of edaphic stress at our sites, the outcome of competition predicted by theory would be a single species dominating each niche. If this is the mechanism behind species distributions seen at our sites, the overlap in realized edaphic niche among species may reflect that the key determinant in these shared edaphic niches (as defined by our sampled properties) is ultimately the stress tolerance of their competitors. However, our field sampling cannot confirm this is the case at our sites, and it could also be due to founder effects, competition, or other biotic effects, or some abiotic factor(s) not addressed here.

Overall, our PCA results suggest that, despite the affinity of these species for vertic clay soils, edaphic factors alone did not drive the sorting of these species into their distinctive mosaic pattern of realized edaphic niches. We found that soil properties drove some differences in realized niche (indeed, much more for some species, e.g., *L. jaredii* subsp. *jaredii*), but we still saw some overlap among species in all analyses. While we likely excluded some essential soil properties for the edaphic niches of these species, the degree of overlap and stressful nature of these soils leaves open the possibility that biotic interaction was influential for the realized niche of these species. We therefore turn to our pot study results to consider fundamental niche and competition from *B. rubens*.

In our pot study results, our model's significant three‐way interaction of soil treatment, competition treatment, and species supports our hypothesis that species would differ in how competition from *B. rubens* altered their response to the gradient of stress and edaphic properties in our soil treatments. While we did not test the performance of *B. rubens* on the treatment soils alone (so cannot address the influence of soil treatments on *B. rubens* without the influence of competition from our study species), these results do suggest that edaphic stress influences these species' competitive ability and that the edaphic heterogeneity at our site leads to heterogeneous outcomes in the soils.

The results of our post hoc contrasts of biomass between soils when competition was absent (Figure 6; Appendix S5) show that within the range of properties captured in our treatment soils, for nearly all species, at least one treatment soil was closer to their true fundamental edaphic niche. While most species had fundamental niche optima that converged on our somewhat sodic treatment soil, these results support our hypothesis that some fundamental edaphic niche optimum existed for these species, at least in terms of those properties that varied among treatment soils (i.e., CEC, N, P, K, Ca, and Mg content). However, our results did not show differences in that optimum among species as much as expected; only *E*. “succulenta” and *L. calliopsidea* had different apparent fundamental niche optima than the other species.

Post hoc contrasts of species’ biomass between competition treatments on the three treatment soils partially support our hypothesis that competition from *B. rubens* would reduce performance of our study species as it does for many native annual forbs (e.g., Salo et al., 2005; Corbin et al., 2007), but not in all cases. These contrasts also partially support our hypothesis that competition's effect would vary by soil treatment, resulting in niche shifts. Competition nearly always reduced biomass for our study species, and while most species converged on the somewhat sodic soil for their fundamental edaphic niche optimum (i.e., when competition was absent), when competition was present, that pattern was weaker: log‐transformed biomass remained significantly higher on the somewhat sodic soil than the non‐sodic and sodic soils for only four species (Figure 6; for post hoc tests, see Appendix S5). We saw significant differences indicative of niche shifts for *L. jaredii* subsp. *jaredii*, *M. radiata*, *M. stricta*, *L. calliopsidea*, and *P. ciliata*. Overall, our pot study results suggest that the realized niches of our study species are influenced by the interaction of edaphic properties and competition, though some species appear to be separated by their fundamental niches, and other factors are likely important to this community's assembly.

The niche shift seen for some species when competition is present (*L. jaredii* subsp. *jaredii*, *M. radiata*, *M. stricta*, *L. calliopsidea*, and *P. ciliata*) indicates some of these species may have a broader fundamental niche than suggested by their restricted realized niche and the well‐defined mosaic pattern at our sites. In our pot study, several species exhibited niche shifts from a common fundamental niche optimum on the somewhat sodic soil to realized niche optima, which varied by species over all three soils. While a species’ realized niche will be inherently smaller than its fundamental niche, there is still the question of what proportion and area within the fundamental niche space that realized niche occupies. If the realized niche optimum differs from the fundamental niche optimum—as we found for several of our species within the edaphic niche space in pot soils—a higher minimum fundamental niche space will be required than if no shift in niche optimum occurred. By this reasoning, our results do suggest that some of our study species have a larger minimum fundamental niche than their restricted distributions and edaphic affinity might initially suggest. If so, “escape” from competition via niche shifts in a heterogeneous environment is plausible for those species (i.e., heterogeneity providing a buffer to invasion; Davies et al., 2005), and climate change may not necessarily mean the reduction in realized niche that one might assume (Emery et al., 2012). Although our results do not distinguish between these possibilities, the persistence of our study species despite extensive invasion by *B. rubens* highlights the need for research on their fundamental versus realized niches to inform conservation and management of this unique and imperiled system. Exploring the performance of *B. rubens* and other species invasive within this system over different soils in the absence of competition from native species could also clarify the degree to which competitors’ stress tolerance influences the realized niches for the species within this community.

The convergence of several of the study species on a fundamental niche optimum at the somewhat sodic soil means that despite niche differences seen among the home soils for species in our PCAs, these species’ fundamental niches in terms of our treatment soils still overlapped more than expected at the somewhat sodic soil. However, our pot study included only three soil treatments, which did not encompass the full range of physical and chemical properties and stress conditions present at our field sites. Moreover, these soils were not exact representations of any single species’ home soil. It is also possible that for species where differences in biomass among soils were weaker when competition was present, the realized niche optimum was simply not captured within our treatment soils. However, those cases may also simply reflect a much‐reduced niche optimum given the limited number of replicates possible in our pot study; trends seen in log‐transformed biomass for some species (e.g., *L. jaredii* subsp. *album*) suggest optima may still exist despite differences not meeting our significance threshold (Figure 6; Appendix S5).

Limitations on where we could collect soils for our pot study may also have influenced our results. As research permitting conditions prevented collection of large enough quantities of soil from Carrizo Plain for use in our pot study, we were restricted to vertic clay soils we could collect in bulk from around Cantua Creek. However, these soils spanned a range of properties largely consistent with the range in the home soils of our study species, though P and Na are the exceptions: The non‐sodic soil was notably lower in Na than was typical for all species’ home soils (which all trended high; Appendix S4), but especially so for some of our Carrizo Plain species. In all pot study soils, P content was also lower than typical for the home soils of the Carrizo Plain species (though consistent for Cantua Creek species) and likely contributed to the discrepancies between our field sampling and pot study results. In the PCA of all species, Na, P, and CEC most defined the first, most explanatory principal component, and those properties were important in the site‐specific PCAs as well: The species with realized niches higher in those properties were *E*. “succulenta”, *L. munzii, L. calliopsidea, L. jaredii* subsp. *album, L. jaredii* subsp. *jaredii, M. stricta*, and *P. ciliata*. However, of those six species, three did not have significantly higher biomass on the sodic soil when competition was present in our pot study, despite realized niches apparently closer to the sodic soil. For *L. munzii, L. jaredii* subsp. *jaredii*, and *M. stricta*, biomass on the sodic soil was lower than or not significantly different from biomass on at least one other soil when competition was present. Since all three species, as well as *M. stricta* and *P. ciliata*, are found at Carrizo Plain, it is likely that our results were influenced by this limitation. Our pot study results therefore should not be interpreted as representative of species’ niche optima in situ, but rather as niche optima in terms of the soil stressors and properties that varied among our treatment soils.

We also note that while vertic clay soils are an edaphically stressful substrate in the field, our pot study entailed a greater water supply relative to soils at our field sites. Since we anticipated more rapid drainage and drying due to the small pot volume, all plants were grown in subirrigated pots, and the total water supply over time was likely greater than the water supply from the typical seasonal precipitation at our field sites. It is therefore possible higher water supply to the soils altered outcomes in competition somewhat, particularly given evidence that reduced water limits *B. rubens* performance and reduces its competitive ability relative to native annuals in similar biomes (Hunter, 1991). However, since the water dynamics of smectite clay and high‐clay soils mean water taken up by the soil may not directly translate to the water available to plants (Kirkham, 2004; Rajakaruna and Boyd, 2008; Velde and Barré, 2010), it is difficult to estimate the effect of this difference.

There are likely other factors at play in this community. Coexistence among these species is likely affected by temporal variation in this community that is not captured in our study, particularly different germination rates and varied precipitation between years. Addressing temporal variation in future work with this system is important because *B. rubens* produces relatively short‐lived seeds that are not capable of deep physiological dormancy (Baskin and Baskin, 2004; Salo, 2004; Jurand et al., 2013), whereas many annuals native to arid regions build up a persistent seed bank in the soil and produce seeds that can remain in deep physiological dormancy for many years (Pake and Venable, 1996). Given these facts and the sensitivity of *B. rubens* to low precipitation and interannual variation in precipitation, some competition and invasion dynamics relating to temporal variability likely were not captured in this study. Future work on how the seed longevity and climatic niche of *B. rubens* influence community dynamics in this system will contribute substantially to the management of this and other edaphically defined plant communities.

## CONCLUSIONS

This study examined how the effects of edaphic stress and competition on species contributed to the community assembly of a suite of annual plants found on vertic clay soils in the San Joaquin Desert—a harsh substrate that has been understudied both in general and in the context of plant community ecology and edaphic endemism. The native annual plant species associated with vertic clay soils at our study sites exhibit a distinctive mosaic pattern in nearly monospecific patches corresponding to species’ realized niches. We found that those realized niches were not entirely distinguished by edaphic properties and that these species exhibit different niche optima across a range of stress‐related properties in vertic clay soils from the region. We also found that competition from an invasive annual grass reduced biomass for nearly all of the native species, but that competition often led to niche shifts within the soils examined. Additionally, our results suggest these species may have broader fundamental niches than their restricted realized niches suggest. These results are consistent with the outcomes predicted by contemporary niche theory, which may have implications for coexistence and community assembly in other heterogeneous, abiotically stressful systems. Our results are also of interest in that many of our study species are rare and endemic to clay soils. The niche shifts observed in our study suggest that narrow realized niches are not necessarily indicative of similarly narrow fundamental niches. This is especially relevant to conservation work on rare, endemic, and otherwise abiotically restricted species, as fundamental niche breadth is important in predicting how species might respond to climate change and related abiotic changes. Lastly, our study examined the community ecology of a flora restricted to vertic clay soil, which we present as a new study system ideal for exploring coexistence dynamics and community assembly on a high‐stress and heterogeneous substrate.

## AUTHOR CONTRIBUTIONS

E.R.F., R.O., and N.R. conceived the ideas and designed methodology with input from D.G.; E.R.F. and R.O. set up and conducted the pot study; E.R.F., A.S., S.M., and R.O. collected the field data; E.R.F., A.S., and S.M. collected the pot study data; E.R.F. and D.G. analyzed the data and interpreted results with input from R.O. and N.R.; E.R.F. led the writing of the manuscript. All authors contributed critically to the drafts and gave final approval for publication.

## Supporting information


**Appendix S1.** Results of principal component analysis for soil samples collected from species patches at both study sites. (A) Biplot of species' soil samples shown over PC1 and PC3. PC1 explains 32.2% of variation; PC3 explains 18.6% of variation. (B) Biplot of species' soil samples shown over PC2 and PC3; values higher on PC2 correspond to soils higher in Mg but with lower Ca and COLE. See Table 2 for loading values.

**Appendix S2.** Results of principal component analysis for soil samples collected from species patches at Cantua Creek. (A) Biplot of species' soil samples shown over PC1 and PC3. PC1 explains 33.0% of variation; PC3 explains 14.8% of variation. (B) Biplot of species' soil samples shown over PC2 and PC3; PC2 explains 24.4% of variation. See Table 2 for loading values.

**Appendix S3.** Results of principal component analysis for soil samples collected from species patches at Carrizo Plain. (A) Biplot of species' soil samples shown over PC1 and PC3. PC1 explains 40.5% of variation; PC3 explains 11.5% of variation. (B) Biplot of species' soil samples shown over PC2 and PC3; PC2 explains 20.9% of variation. See Table 2 for loading values.

**Appendix S4**: Soil properties of soils from “home soil” patches for each species. CC, Cantua Creek; CP, Carrizo Plain.

**Appendix S5.** Post hoc contrasts of species by soil treatments across competition treatments. Results were calculated using Tukey's honest significant difference method to find differences while controlling the family‐wise error rate. Differences in estimated marginal means (Estimate) were found using log‐transformed biomass modeled as log(Biomass *+* 1) *~* Species ∗ Soil ∗ Treatment + Block + (1 | Block:Soil) (Table 4) averaged over blocks. df = 189, confidence level = 0.95 in all cases. C, competition present; NC, competition absent; NS, non‐sodic soil; SS, somewhat sodic; S, sodic. Differences in estimated marginal means listed are log‐transformed.

**Appendix S6.** Post hoc contrasts of species by competition treatment across soil treatments. Contrasts run as Competition absent – Competition present. Results were calculated using Tukey's honest significant difference method. Differences in estimated marginal means (Estimate) were found using log‐transformed biomass modeled as log(Biomass + 1) ~Species * Soil * Treatment + Block + (1 | Block:Soil) (Table 4), averaged over blocks. df = 201, confidence level = 0.95 in all cases. NS, non‐sodic soil; SS, somewhat sodic; S, sodic. Differences in estimated marginal means listed are log‐transformed.

## Data Availability

Supporting data are publicly available online at Dryad: https://doi.org/10.5061/dryad.ffbg79d67.
