# Supplementary material for: Soil, competition, and niche shifts shape the floral mosaic of an annual plant diversity hotspot
Source: Am J Bot. 2026 Mar 5;113(3):e70171. doi: 10.1002/ajb2.70171 (PMC13003719; doi:10.1002/ajb2.70171)

**Appendix S1.** Results of principal component analysis for soil samples collected from species patches at both study sites. (A) Biplot of species’ soil samples shown over PC1 and PC3. PC1 explains 32.2% of variation; PC3 explains 18.6% of variation. (B) Biplot of species’ soil samples shown over PC2 and PC3; values higher on PC2 correspond to soils higher in Mg but with lower Ca and COLE. See Table 2 for loading values.


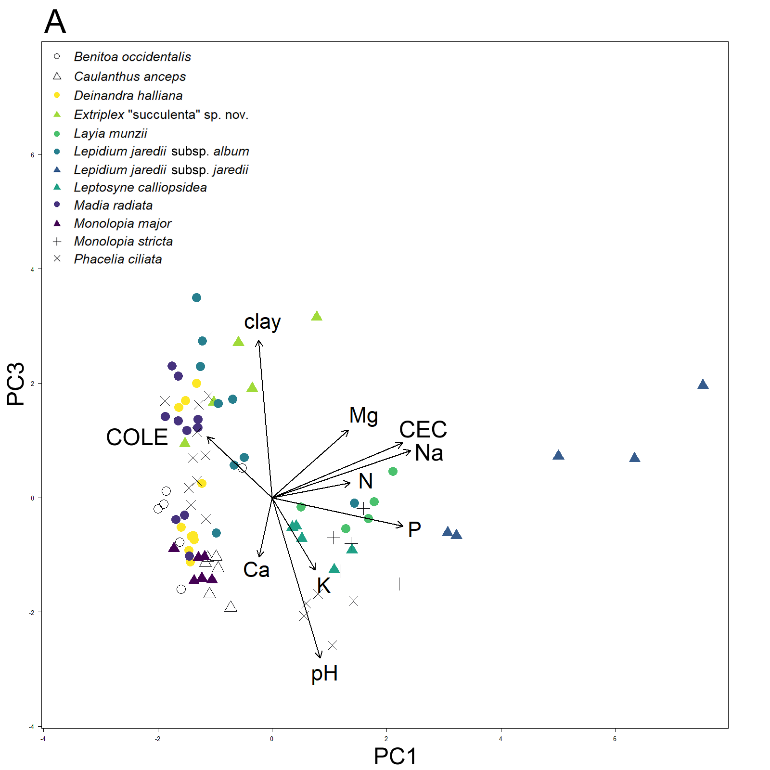

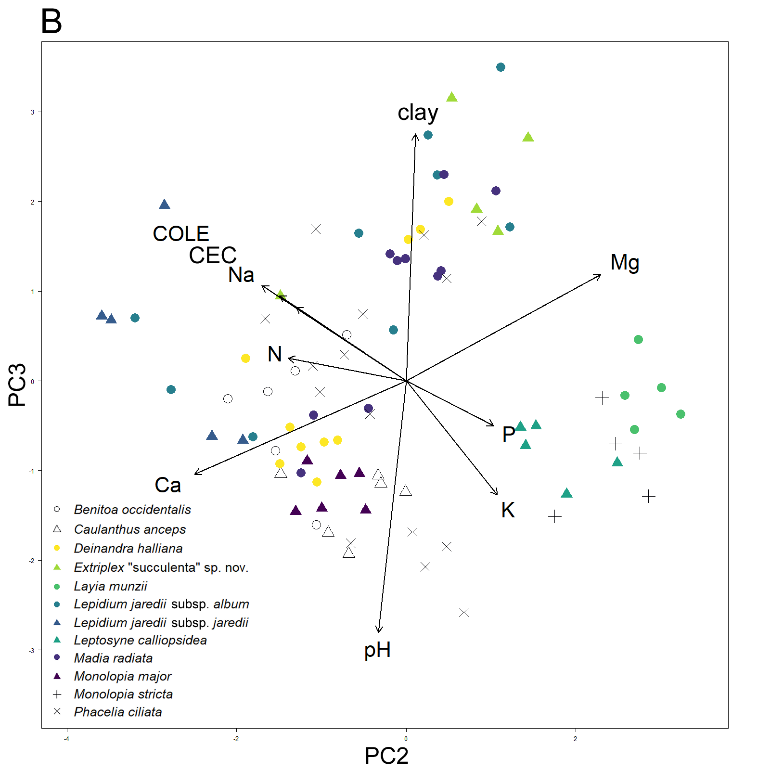

Supplement: Supplementary file 1 — Appendix S1. Results of principal component analysis for soil samples collected from species patches at both study sites. (A) Biplot of species' soil samples shown over PC1 and PC3. PC1 explains 32.2% of variation; PC3 explains 18.6% of variation. (B) Biplot of species' soil samples shown over PC2 and PC3; values higher on PC2 correspond to soils higher in Mg but with lower Ca and COLE. See Table 2 for loading values. [file AJB2-113-e70171-s006.docx]
