# Supplementary material for: Soil, competition, and niche shifts shape the floral mosaic of an annual plant diversity hotspot
Source: Am J Bot. 2026 Mar 5;113(3):e70171. doi: 10.1002/ajb2.70171 (PMC13003719; doi:10.1002/ajb2.70171)

**Appendix S2.** Results of principal component analysis for soil samples collected from species patches at Cantua Creek. (A) Biplot of species’ soil samples shown over PC1 and PC3. PC1 explains 33.0% of variation; PC3 explains 14.8% of variation. (B) Biplot of species’ soil samples shown over PC2 and PC3; PC2 explains 24.4% of variation. See Table 2 for loading values.
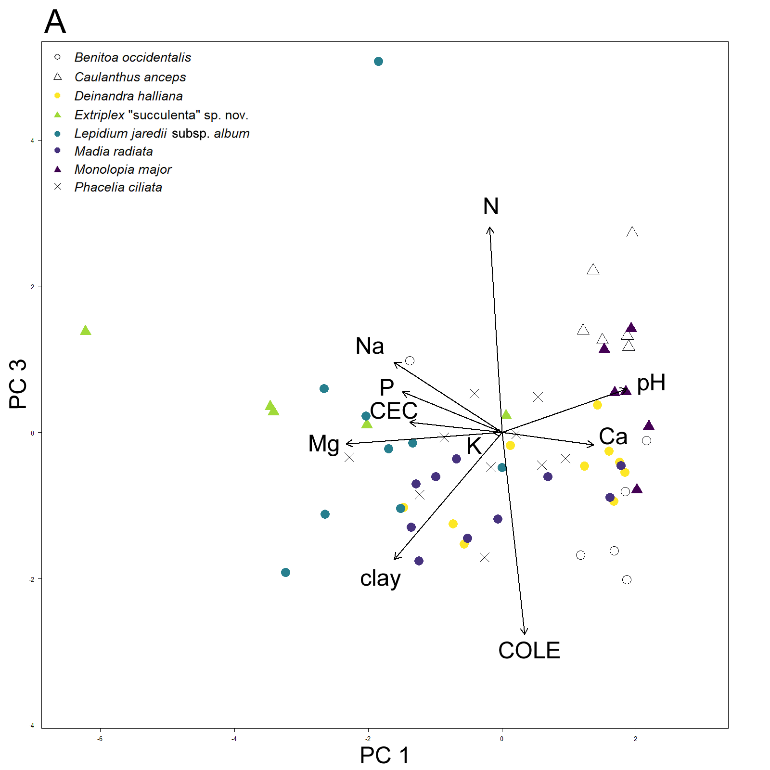

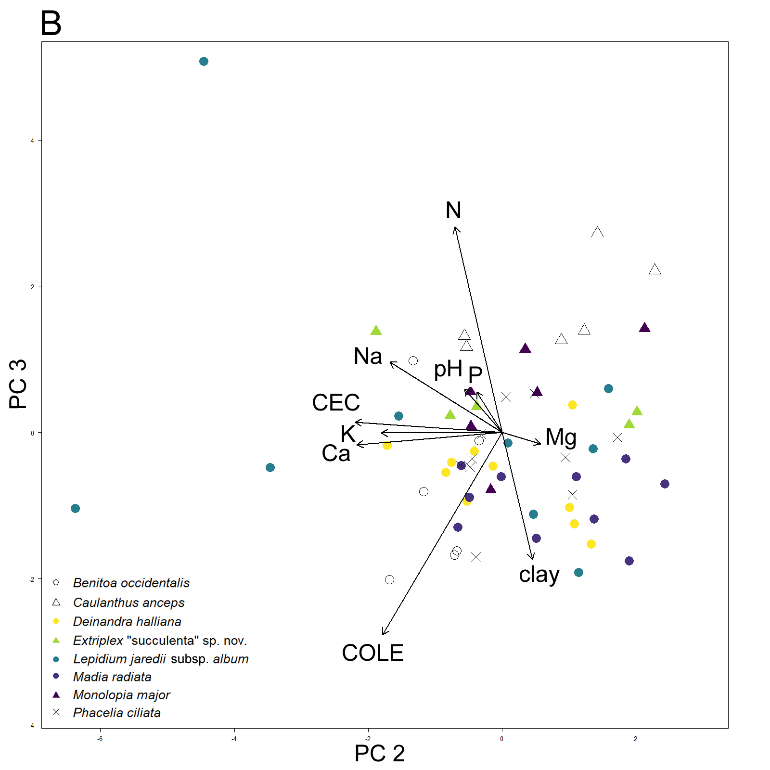

Supplement: Supplementary file 2 — Appendix S2. Results of principal component analysis for soil samples collected from species patches at Cantua Creek. (A) Biplot of species' soil samples shown over PC1 and PC3. PC1 explains 33.0% of variation; PC3 explains 14.8% of variation. (B) Biplot of species' soil samples shown over PC2 and PC3; PC2 explains 24.4% of variation. See Table 2 for loading values. [file AJB2-113-e70171-s004.docx]
