# Supplementary material for: Soil, competition, and niche shifts shape the floral mosaic of an annual plant diversity hotspot
Source: Am J Bot. 2026 Mar 5;113(3):e70171. doi: 10.1002/ajb2.70171 (PMC13003719; doi:10.1002/ajb2.70171)

**Appendix S3.** Results of principal component analysis for soil samples collected from species patches at Carrizo Plain. (A) Biplot of species’ soil samples shown over PC1 and PC3. PC1 explains 40.5% of variation; PC3 explains 11.5% of variation. (B) Biplot of species’ soil samples shown over PC2 and PC3; PC2 explains 20.9% of variation. See Table 2 for loading values.
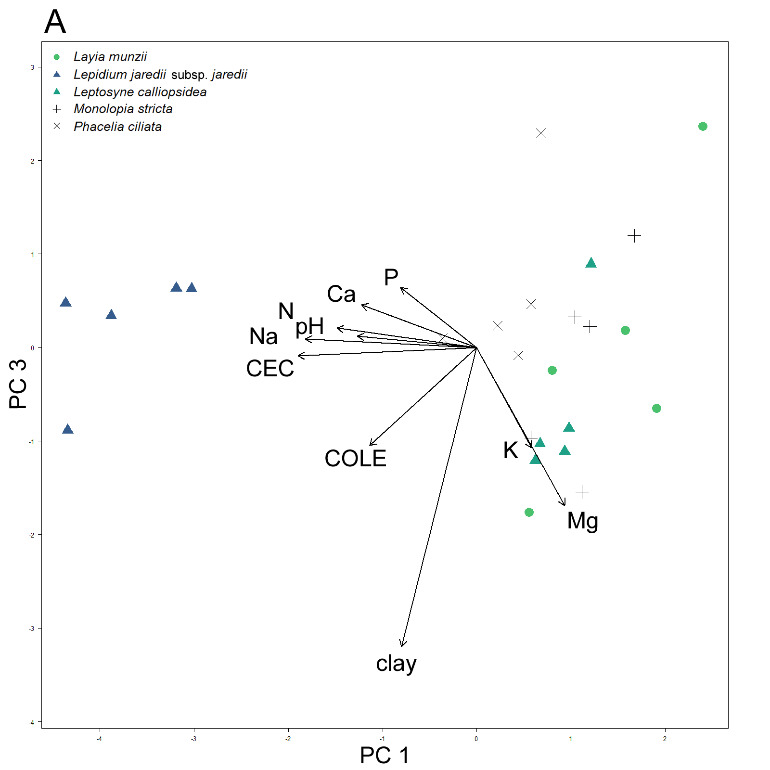

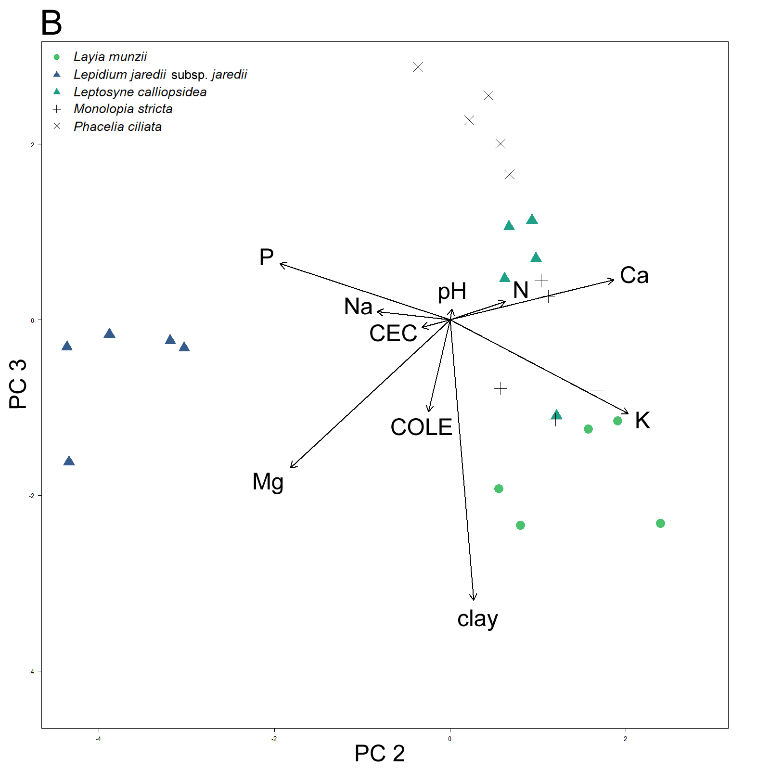

Supplement: Supplementary file 3 — Appendix S3. Results of principal component analysis for soil samples collected from species patches at Carrizo Plain. (A) Biplot of species' soil samples shown over PC1 and PC3. PC1 explains 40.5% of variation; PC3 explains 11.5% of variation. (B) Biplot of species' soil samples shown over PC2 and PC3; PC2 explains 20.9% of variation. See Table 2 for loading values. [file AJB2-113-e70171-s002.docx]
