# Supplementary material for: Soil, competition, and niche shifts shape the floral mosaic of an annual plant diversity hotspot
Source: Am J Bot. 2026 Mar 5;113(3):e70171. doi: 10.1002/ajb2.70171 (PMC13003719; doi:10.1002/ajb2.70171)
Supplement: Supplementary file 4 — Appendix S4: Soil properties of soils from “home soil” patches for each species. CC, Cantua Creek; CP, Carrizo Plain. [file AJB2-113-e70171-s003.docx]

**Appendix S4**. Soil properties of soils from “home soil” patches for each species. CC, Cantua Creek; CP, Carrizo Plain.

|  |  |  | N | P | K | Ca | Mg | Na | CEC |  |  | Clay |  |
| --- | --- | --- | --- | --- | --- | --- | --- | --- | --- | --- | --- | --- | --- |
| Species | Site | pH | ppm, all elements | | | | | | meq/100g | Ca:Mg | Na:K | % | COLE |
| *Benitoa occidentalis* | CC | 6.5 | 9 | 26 | 551 | 6147 | 588 | 615 | 42.8 | 6.34 | 1.90 | 53% | 0.18 |
|  | CC | 7.5 | 3 | 4 | 488 | 6779 | 139 | 23 | 36.3 | 29.58 | 0.08 | 49% | 0.28 |
|  | CC | 7.5 | 2 | 9 | 494 | 5915 | 233 | 33 | 32.8 | 15.40 | 0.11 | 55% | 0.25 |
|  | CC | 7.6 | 3 | 7 | 495 | 5572 | 181 | 26 | 30.7 | 18.67 | 0.09 | 31% | 0.23 |
|  | CC | 7.7 | 2 | 1 | 512 | 6547 | 112 | 311 | 36.2 | 35.45 | 1.03 | 47% | 0.23 |
|  | CC | 7.6 | 2 | 4 | 450 | 6400 | 161 | 20 | 34.5 | 24.11 | 0.08 | 53% | 0.25 |
| *Caulanthus anceps* | CC | 7.8 | 18 | 4 | 530 | 5491 | 128 | 35 | 30.0 | 26.02 | 0.11 | 43% | 0.21 |
|  | CC | 7.6 | 8 | 8 | 519 | 4767 | 150 | 36 | 26.5 | 19.27 | 0.12 | 43% | 0.17 |
|  | CC | 7.9 | 16 | 9 | 400 | 4762 | 212 | 15 | 26.6 | 13.62 | 0.06 | 31% | 0.16 |
|  | CC | 7.8 | 13 | 13 | 413 | 3793 | 195 | 14 | 21.6 | 11.80 | 0.06 | 41% | 0.16 |
|  | CC | 7.6 | 8 | 7 | 568 | 5809 | 140 | 39 | 31.8 | 25.16 | 0.12 | 35% | 0.19 |
|  | CC | 7.6 | 8 | 9 | 435 | 4458 | 186 | 455 | 26.9 | 14.54 | 1.78 | 41% | 0.17 |
| *Deinandra halliana* | CC | 7.7 | 6 | 9 | 390 | 5381 | 194 | 54 | 29.7 | 16.82 | 0.24 | 48% | 0.19 |
|  | CC | 5.8 | 5 | 9 | 425 | 7562 | 369 | 37 | 51.9 | 12.43 | 0.15 | 38% | 0.22 |
|  | CC | 7.0 | 5 | 10 | 494 | 4018 | 742 | 130 | 28.0 | 3.28 | 0.45 | 70% | 0.22 |
|  | CC | 7.0 | 3 | 10 | 420 | 4209 | 791 | 116 | 29.1 | 3.23 | 0.47 | 66% | 0.23 |
|  | CC | 6.5 | 4 | 9 | 523 | 4092 | 892 | 38 | 31.6 | 2.78 | 0.12 | 70% | 0.20 |
|  | CC | 8.1 | 7 | 8 | 495 | 5747 | 186 | 43 | 31.7 | 18.74 | 0.15 | 51% | 0.22 |
| *Deinandra halliana* | CC | 8.1 | 5 | 7 | 484 | 5813 | 178 | 29 | 31.8 | 19.80 | 0.10 | 53% | 0.24 |
|  | CC | 8.2 | 5 | 6 | 491 | 6052 | 120 | 225 | 33.4 | 30.58 | 0.78 | 49% | 0.23 |
|  | CC | 8.2 | 5 | 7 | 549 | 5263 | 189 | 231 | 30.2 | 16.89 | 0.72 | 45% | 0.23 |
|  | CC | 8.1 | 4 | 14 | 473 | 5435 | 224 | 149 | 30.8 | 14.71 | 0.54 | 51% | 0.22 |
| *Extriplex* “succulenta" sp. nov. | CC | 5.0 | 14 | 14 | 468 | 5745 | 331 | 41 | 38.5 | 10.53 | 0.15 | 36% | 0.24 |
|  | CC | 5.8 | 8 | 12 | 416 | 2388 | 1544 | 892 | 36.5 | 0.94 | 3.65 | 62% | 0.18 |
|  | CC | 5.7 | 11 | 13 | 466 | 3468 | 1824 | 3290 | 60.5 | 1.15 | 12.01 | 62% | 0.19 |
|  | CC | 5.8 | 5 | 18 | 551 | 3833 | 1359 | 1092 | 45.0 | 1.71 | 3.37 | 55% | 0.19 |
|  | CC | 6.5 | 6 | 19 | 471 | 2799 | 957 | 529 | 27.4 | 1.77 | 1.91 | 62% | 0.19 |
| *Leptosyne calliopsidea* | CP | 7.7 | 6 | 50 | 741 | 4320 | 1273 | 199 | 34.8 | 2.06 | 0.46 | 51% | 0.15 |
|  | CP | 7.8 | 7 | 40 | 924 | 4406 | 1355 | 286 | 36.7 | 1.97 | 0.53 | 51% | 0.15 |
|  | CP | 8.0 | 3 | 49 | 735 | 4016 | 1408 | 612 | 36.2 | 1.73 | 1.42 | 52% | 0.16 |
|  | CP | 7.8 | 5 | 105 | 774 | 2004 | 1417 | 1909 | 31.9 | 0.86 | 4.19 | 41% | 0.16 |
|  | CP | 8.0 | 7 | 70 | 1101 | 4380 | 1249 | 162 | 35.6 | 2.13 | 0.25 | 53% | 0.13 |
| *Lepidium jaredii* subsp*. album* | CC | 5.9 | 7 | 10 | 457 | 3758 | 708 | 536 | 33.8 | 3.22 | 1.99 | 67% | 0.19 |
|  | CC | 5.7 | 5 | 17 | 468 | 3919 | 952 | 411 | 38.4 | 2.50 | 1.49 | 67% | 0.22 |
|  | CC | 5.9 | 6 | 16 | 458 | 2920 | 1251 | 711 | 35.1 | 1.42 | 2.64 | 53% | 0.18 |
|  | CC | 5.5 | 3 | 14 | 488 | 2483 | 1434 | 451 | 37.0 | 1.05 | 1.57 | 69% | 0.24 |
|  | CC | 6.6 | 12 | 10 | 431 | 4773 | 869 | 581 | 36.8 | 3.33 | 2.29 | 61% | 0.22 |
|  | CC | 7.9 | 8 | 18 | 609 | 8111 | 282 | 2941 | 57.1 | 17.44 | 8.21 | 65% | 0.29 |
|  | CC | 7.9 | 3 | 15 | 579 | 6302 | 210 | 1926 | 43.0 | 18.20 | 5.66 | 47% | 0.25 |
| *Lepidium jaredii* subsp*. album* | CC | 6.7 | 4 | 32 | 605 | 5080 | 685 | 1015 | 38.7 | 4.50 | 2.85 | 53% | 0.21 |
|  | CC | 7.6 | 45 | 27 | 611 | 6726 | 559 | 1968 | 48.3 | 7.30 | 5.48 | 51% | 0.20 |
| *Lepidium jaredii* subsp*. jaredii* | CP | 8.2 | 28 | 108 | 487 | 6018 | 636 | 16638 | 109.0 | 5.74 | 58.10 | 51% | 0.15 |
|  | CP | 7.9 | 27 | 103 | 492 | 6366 | 658 | 13588 | 97.5 | 5.87 | 46.97 | 49% | 0.21 |
|  | CP | 7.9 | 34 | 125 | 497 | 4876 | 1610 | 18004 | 117.0 | 1.84 | 61.61 | 54% | 0.17 |
|  | CP | 8.2 | 8 | 138 | 559 | 7254 | 359 | 8027 | 75.5 | 12.25 | 24.42 | 49% | 0.21 |
|  | CP | 8.3 | 8 | 131 | 626 | 6688 | 553 | 8287 | 75.6 | 7.33 | 22.51 | 47% | 0.21 |
| *Layia munzii* | CP | 7.7 | 6 | 114 | 922 | 1584 | 2358 | 2864 | 42.1 | 0.41 | 5.28 | 49% | 0.22 |
|  | CP | 7.6 | 3 | 105 | 667 | 3090 | 1842 | 450 | 34.2 | 1.02 | 1.15 | 45% | 0.14 |
|  | CP | 7.4 | 3 | 90 | 46 | 1163 | 1502 | 1438 | 24.5 | 0.47 | 53.16 | 36% | 0.13 |
|  | CP | 8.0 | 4 | 116 | 592 | 1323 | 2156 | 2122 | 35.1 | 0.37 | 6.10 | 45% | 0.18 |
|  | CP | 7.3 | 2 | 123 | 882 | 2830 | 1784 | 843 | 34.7 | 0.96 | 1.63 | 50% | 0.13 |
| *Monolopia major* | CC | 7.9 | 7 | 8 | 547 | 5648 | 128 | 53 | 30.9 | 26.76 | 0.16 | 41% | 0.20 |
|  | CC | 8.0 | 7 | 9 | 523 | 4984 | 186 | 223 | 28.7 | 16.25 | 0.73 | 42% | 0.18 |
|  | CC | 8.0 | 8 | 1 | 348 | 5220 | 109 | 27 | 27.9 | 29.04 | 0.13 | 49% | 0.15 |
|  | CC | 8.1 | 6 | 4 | 488 | 5899 | 166 | 55 | 32.3 | 21.55 | 0.19 | 40% | 0.22 |
|  | CC | 7.9 | 7 | 7 | 468 | 5228 | 179 | 57 | 29.0 | 17.71 | 0.21 | 45% | 0.20 |
|  | CC | 7.9 | 3 | 4 | 476 | 5575 | 153 | 27 | 30.4 | 22.10 | 0.10 | 45% | 0.23 |
| *Madia radiata* | CC | 5.9 | 3 | 8 | 479 | 2900 | 720 | 39 | 26.2 | 2.44 | 0.14 | 65% | 0.19 |
|  | CC | 6.5 | 4 | 12 | 431 | 4190 | 429 | 47 | 27.8 | 5.92 | 0.19 | 61% | 0.22 |
|  | CC | 6.6 | 2 | 15 | 489 | 4434 | 784 | 53 | 32.0 | 3.43 | 0.18 | 61% | 0.19 |
| *Madia radiata* | CC | 6.8 | 3 | 17 | 445 | 4065 | 448 | 81 | 26.2 | 5.50 | 0.31 | 65% | 0.19 |
|  | CC | 6.2 | 1 | 9 | 388 | 3639 | 954 | 150 | 31.4 | 2.31 | 0.66 | 65% | 0.23 |
|  | CC | 8.1 | 4 | 8 | 503 | 5961 | 180 | 42 | 32.7 | 20.08 | 0.14 | 47% | 0.22 |
|  | CC | 6.9 | 3 | 15 | 512 | 4455 | 591 | 47 | 29.0 | 4.57 | 0.16 | 65% | 0.23 |
|  | CC | 6.9 | 4 | 24 | 629 | 4633 | 681 | 43 | 31.0 | 4.13 | 0.12 | 67% | 0.23 |
|  | CC | 7.6 | 5 | 2 | 530 | 5448 | 244 | 45 | 30.7 | 13.54 | 0.14 | 49% | 0.24 |
|  | CC | 7.4 | 2 | 16 | 531 | 4959 | 324 | 107 | 29.2 | 9.28 | 0.34 | 49% | 0.22 |
| *Monolopia stricta* | CP | 7.9 | 7 | 91 | 786 | 1959 | 1512 | 2762 | 36.2 | 0.79 | 5.98 | 51% | 0.16 |
|  | CP | 7.8 | 2 | 86 | 1125 | 5850 | 1789 | 1770 | 54.5 | 1.98 | 2.68 | 44% | 0.12 |
|  | CP | 7.8 | 4 | 112 | 794 | 2304 | 1561 | 1082 | 31.1 | 0.90 | 2.32 | 45% | 0.15 |
|  | CP | 7.9 | 2 | 96 | 693 | 2530 | 1384 | 940 | 29.9 | 1.11 | 2.31 | 43% | 0.11 |
|  | CP | 7.7 | 4 | 82 | 1169 | 3342 | 1614 | 391 | 34.6 | 1.26 | 0.57 | 51% | 0.17 |
| *Phacelia ciliata* | CC | 6.0 | 3 | 7 | 468 | 5745 | 331 | 41 | 38.5 | 10.53 | 0.15 | 61% | 0.24 |
|  | CC | 6.1 | 4 | 28 | 554 | 3529 | 836 | 85 | 30.5 | 2.56 | 0.26 | 63% | 0.20 |
|  | CC | 6.7 | 7 | 11 | 452 | 3771 | 966 | 216 | 30.2 | 2.37 | 0.81 | 61% | 0.23 |
|  | CC | 6.7 | 6 | 19 | 469 | 3703 | 603 | 40 | 26.0 | 3.72 | 0.15 | 61% | 0.19 |
|  | CC | 6.6 | 11 | 21 | 485 | 4939 | 301 | 33 | 30.3 | 9.95 | 0.12 | 57% | 0.20 |
|  | CC | 6.5 | 10 | 20 | 390 | 6610 | 128 | 14 | 37.9 | 31.32 | 0.06 | 55% | 0.22 |
|  | CC | 7.1 | 8 | 16 | 527 | 5656 | 251 | 21 | 31.7 | 13.67 | 0.07 | 53% | 0.23 |
|  | CC | 7.0 | 4 | 23 | 501 | 5612 | 426 | 273 | 34.0 | 7.99 | 0.93 | 53% | 0.22 |
|  | CC | 7.6 | 9 | 21 | 569 | 4668 | 204 | 50 | 26.6 | 13.88 | 0.15 | 53% | 0.20 |
| *Phacelia ciliata* | CC | 7.3 | 8 | 9 | 519 | 5460 | 357 | 42 | 31.7 | 9.28 | 0.14 | 49% | 0.23 |
|  | CP | 7.7 | 10 | 66 | 1121 | 5760 | 491 | 63 | 35.9 | 7.11 | 0.10 | 46% | 0.15 |
|  | CP | 7.9 | 16 | 78 | 1102 | 5072 | 444 | 71 | 32.1 | 6.93 | 0.11 | 38% | 0.13 |
|  | CP | 7.6 | 30 | 61 | 1430 | 6362 | 579 | 92 | 40.6 | 6.66 | 0.11 | 45% | 0.19 |
|  | CP | 7.9 | 6 | 61 | 1216 | 6618 | 581 | 53 | 41.1 | 6.91 | 0.07 | 46% | 0.16 |
|  | CP | 7.6 | 14 | 53 | 1197 | 6560 | 591 | 69 | 41.0 | 6.73 | 0.10 | 49% | 0.15 |
