# Supplementary material for: Soil, competition, and niche shifts shape the floral mosaic of an annual plant diversity hotspot
Source: Am J Bot. 2026 Mar 5;113(3):e70171. doi: 10.1002/ajb2.70171 (PMC13003719; doi:10.1002/ajb2.70171)
Supplement: Supplementary file 5 — Appendix S5. Post hoc contrasts of species by soil treatments across competition treatments. Results were calculated using Tukey's honest significant difference method to find differences while controlling the family‐wise error rate. Differences in estimated marginal means (Estimate) were found using log‐transformed biomass modeled as log(Biomass + 1) ~ Species ∗ Soil ∗ Treatment + Block + (1 | Block:Soil) (Table 4) averaged over blocks. df = 189, confidence level = 0.95 in all cases. C, competition present; NC, competition absent; NS, non‐sodic soil; SS, somewhat sodic; S, sodic. Differences in estimated marginal means listed are log‐transformed. [file AJB2-113-e70171-s005.docx]

**Appendix S5.** Post hoc contrasts of species by soil treatments across competition treatments. Results were calculated using Tukey's honest significant difference method to find differences while controlling the family-wise error rate. Differences in estimated marginal means (Estimate) were found using log-transformed biomass modeled as log (Biomass *+* 1) *~* Species * Soil * Treatment + Block + (1 | Block:Soil) (Table 4) averaged over blocks. df = 189, confidence level = 0.95 in all cases. C, competition present; NC, competition absent; NS, non-sodic soil; SS, somewhat sodic; S, sodic. Differences in estimated marginal means listed are log-transformed.

| Species | **Treatment** | **Contrast** | **Estimate** | **SE** | ***t*-ratio** | ***P*** |
| --- | --- | --- | --- | --- | --- | --- |
| *Benitoa occidentalis* | NC | NS - SS | -0.297 | 0.163 | -1.820 | 0.166 |
|  |  | NS - S | 0.041 | 0.163 | 0.249 | 0.966 |
|  |  | SS - S | 0.338 | 0.163 | 2.069 | 0.099 |
|  | C | NS - SS | -0.368 | 0.163 | -2.253 | 0.065 |
|  |  | NS - S | -0.007 | 0.163 | -0.044 | 0.999 |
|  |  | SS - S | 0.360 | 0.163 | 2.209 | 0.072 |
| *Caulanthus anceps* | NC | NS - SS | -0.461 | 0.176 | -2.618 | 0.026 |
|  |  | NS - S | 0.071 | 0.176 | 0.404 | 0.914 |
|  |  | SS - S | 0.533 | 0.163 | 3.264 | 0.004 |
|  | C | NS - SS | -0.198 | 0.163 | -1.213 | 0.447 |
|  |  | NS - S | 0.363 | 0.163 | 2.223 | 0.070 |
|  |  | SS - S | 0.561 | 0.163 | 3.436 | 0.002 |
| *Deinandra halliana* | NC | NS - SS | -0.775 | 0.163 | -4.747 | <0.001 |
|  |  | NS - S | 0.061 | 0.163 | 0.376 | 0.925 |
|  |  | SS - S | 0.836 | 0.163 | 5.124 | <0.001 |
|  | C | NS - SS | -0.878 | 0.163 | -5.383 | <0.001 |
|  |  | NS - S | 0.105 | 0.163 | 0.646 | 0.795 |
|  |  | SS - S | 0.984 | 0.163 | 6.029 | <0.001 |
| *Extriplex* "succulenta" sp. nov. | NC | NS - SS | 0.334 | 0.163 | 2.046 | 0.104 |
|  |  | NS - S | 0.002 | 0.163 | 0.012 | 1.000 |
|  |  | SS - S | -0.332 | 0.163 | -2.034 | 0.107 |
|  | C | NS - SS | 0.248 | 0.163 | 1.522 | 0.283 |
|  |  | NS - S | -0.233 | 0.163 | -1.428 | 0.328 |
|  |  | SS - S | -0.481 | 0.163 | -2.950 | 0.010 |
| *Layia munzii* | NC | NS - SS | -0.839 | 0.163 | -5.143 | <0.001 |
|  |  | NS - S | -0.074 | 0.163 | -0.454 | 0.893 |
|  |  | SS - S | 0.765 | 0.163 | 4.689 | <0.001 |
| *Layia munzii* | C | NS - SS | -0.357 | 0.163 | -2.189 | 0.076 |
|  |  | NS - S | 0.237 | 0.163 | 1.450 | 0.317 |
|  |  | SS - S | 0.594 | 0.163 | 3.639 | 0.001 |
| *Lepidium jaredii* subsp. *album* | NC | NS - SS | -1.155 | 0.163 | -7.080 | <0.001 |
|  |  | NS - S | -0.797 | 0.163 | -4.886 | <0.001 |
|  |  | SS - S | 0.358 | 0.163 | 2.194 | 0.075 |
|  | C | NS - SS | -0.400 | 0.163 | -2.450 | 0.040 |
|  |  | NS - S | -0.393 | 0.163 | -2.411 | 0.044 |
|  |  | SS - S | 0.006 | 0.163 | 0.039 | 0.999 |
| *Lepidium jaredii* subsp. *jaredii* | NC | NS - SS | -0.743 | 0.163 | -4.557 | <0.001 |
|  |  | NS - S | -0.511 | 0.163 | -3.133 | 0.006 |
|  |  | SS - S | 0.232 | 0.163 | 1.423 | 0.331 |
|  | C | NS - SS | 0.234 | 0.176 | 1.328 | 0.381 |
|  |  | NS - S | 0.050 | 0.163 | 0.306 | 0.950 |
|  |  | SS - S | -0.184 | 0.176 | -1.045 | 0.549 |
| *Leptosyne calliopsidea* | NC | NS - SS | -0.501 | 0.163 | -3.068 | 0.007 |
|  |  | NS - S | -0.595 | 0.163 | -3.647 | <0.001 |
|  |  | SS - S | -0.094 | 0.163 | -0.579 | 0.832 |
|  | C | NS - SS | 0.623 | 0.176 | 3.533 | 0.001 |
|  |  | NS - S | -0.159 | 0.176 | -0.905 | 0.638 |
|  |  | SS - S | -0.782 | 0.188 | -4.152 | <0.001 |
| *Madia radiata* | NC | NS - SS | -0.620 | 0.163 | -3.800 | <0.001 |
|  |  | NS - S | -0.139 | 0.163 | -0.850 | 0.673 |
|  |  | SS - S | 0.481 | 0.163 | 2.951 | 0.010 |
|  | C | NS - SS | -0.036 | 0.163 | -0.221 | 0.973 |
|  |  | NS - S | 0.455 | 0.163 | 2.786 | 0.016 |
|  |  | SS - S | 0.491 | 0.163 | 3.007 | 0.008 |
| *Monolopia major* | NC | NS - SS | -0.526 | 0.163 | -3.223 | 0.004 |
|  |  | NS - S | -0.112 | 0.163 | -0.689 | 0.770 |
|  |  | SS - S | 0.414 | 0.163 | 2.534 | 0.032 |
|  | C | NS - SS | -0.137 | 0.163 | -0.839 | 0.679 |
|  |  | NS - S | 0.442 | 0.163 | 2.712 | 0.020 |
|  |  | SS - S | 0.579 | 0.163 | 3.551 | 0.001 |
| *Monolopia stricta* | NC | NS - SS | -0.522 | 0.163 | -3.199 | 0.005 |
|  |  | NS - S | 0.098 | 0.163 | 0.600 | 0.820 |
|  |  | SS - S | 0.620 | 0.163 | 3.799 | <0.001 |
|  | C | NS - SS | 0.359 | 0.176 | 2.036 | 0.106 |
|  |  | NS - S | 0.811 | 0.176 | 4.600 | <0.001 |
|  |  | SS - S | 0.452 | 0.163 | 2.769 | 0.017 |
| *Phacelia ciliata* | NC | NS - SS | -0.522 | 0.176 | -2.964 | 0.010 |
|  |  | NS - S | -0.029 | 0.163 | -0.178 | 0.983 |
|  |  | SS - S | 0.493 | 0.176 | 2.799 | 0.016 |
|  | C | NS - SS | 0.225 | 0.163 | 1.382 | 0.353 |
|  |  | NS - S | 0.457 | 0.163 | 2.802 | 0.015 |
|  |  | SS - S | 0.232 | 0.163 | 1.421 | 0.332 |
