# Supplementary material for: Soil, competition, and niche shifts shape the floral mosaic of an annual plant diversity hotspot
Source: Am J Bot. 2026 Mar 5;113(3):e70171. doi: 10.1002/ajb2.70171 (PMC13003719; doi:10.1002/ajb2.70171)
Supplement: Supplementary file 6 — Appendix S6. Post hoc contrasts of species by competition treatment across soil treatments. Contrasts run as Competition absent – Competition present. Results were calculated using Tukey's honest significant difference method. Differences in estimated marginal means (Estimate) were found using log‐transformed biomass modeled as log(Biomass + 1) ~Species * Soil * Treatment + Block + (1 | Block:Soil) (Table 4), averaged over blocks. df = 201, confidence level = 0.95 in all cases. NS, non‐sodic soil; SS, somewhat sodic; S, sodic. Differences in estimated marginal means listed are log‐transformed. [file AJB2-113-e70171-s001.docx]

**Appendix S6.** Post hoc contrasts of species by competition treatment across soil treatments. Contrasts run as Competition absent – Competition present. Results were calculated using Tukey's honest significant difference method. Differences in estimated marginal means (Estimate) were found using log-transformed biomass modeled as log (Biomass + 1) ~ Species * Soil * Treatment + Block + (1 | Block:Soil) (Table 4), averaged over blocks. df = 201, confidence level = 0.95 in all cases. NS, non-sodic soil; SS, somewhat sodic; S, sodic. Differences in estimated marginal means listed are log-transformed.

| Species | **Soil** | **Estimate** | **SE** | ***t*-ratio** | ***P*** |
| --- | --- | --- | --- | --- | --- |
| *Benitoa occidentalis* | NS | 0.574 | 0.160 | 3.589 | < 0.001 |
|  | SS | 0.503 | 0.160 | 3.148 | 0.002 |
|  | S | 0.526 | 0.160 | 3.290 | 0.001 |
| *Caulanthus anceps* | NS | 0.362 | 0.173 | 2.088 | 0.038 |
|  | SS | 0.625 | 0.160 | 3.908 | < 0.001 |
|  | S | 0.653 | 0.160 | 4.084 | < 0.001 |
| *Deinandra halliana* | NS | 1.090 | 0.160 | 6.814 | < 0.001 |
|  | SS | 0.986 | 0.160 | 6.166 | < 0.001 |
|  | S | 1.134 | 0.160 | 7.090 | < 0.001 |
| *Extriplex* "succulenta" sp. nov. | NS | 0.943 | 0.160 | 5.899 | < 0.001 |
|  | SS | 0.858 | 0.160 | 5.363 | < 0.001 |
|  | S | 0.708 | 0.160 | 4.429 | < 0.001 |
| *Layia munzii* | NS | 0.306 | 0.605 | 0.160 | 3.781 |
|  | SS | 0.429 | 1.087 | 0.160 | 6.795 |
|  | S | 0.521 | 0.915 | 0.160 | 5.724 |
| *Lepidium jaredii* subsp. *album* | NS | 0.192 | 0.160 | 1.200 | 0.231 |
|  | SS | 0.947 | 0.160 | 5.924 | < 0.001 |
|  | S | 0.596 | 0.160 | 3.725 | < 0.001 |
| *Lepidium jaredii* subsp. *jaredii* | NS | 0.152 | 0.160 | 0.949 | 0.344 |
|  | SS | 1.129 | 0.173 | 6.518 | < 0.001 |
|  | S | 0.713 | 0.160 | 4.457 | < 0.001 |
| *Leptosyne calliopsidea* | NS | 0.424 | 0.160 | 2.654 | 0.009 |
|  | SS | 1.548 | 0.173 | 8.935 | < 0.001 |
|  | S | 0.860 | 0.173 | 4.965 | < 0.001 |
| *Madia radiata* | NS | 0.073 | 0.189 | 0.160 | 1.183 |
|  | SS | 0.271 | 0.773 | 0.160 | 4.834 |
|  | S | 0.334 | 0.782 | 0.160 | 4.892 |
| *Monolopia major* | NS | 0.416 | 0.160 | 2.600 | 0.010 |
|  | SS | 0.805 | 0.160 | 5.032 | < 0.001 |
|  | S | 0.971 | 0.160 | 6.069 | < 0.001 |
| *Monolopia stricta* | NS | 0.156 | 0.173 | 0.903 | 0.368 |
|  | SS | 1.037 | 0.160 | 6.485 | < 0.001 |
|  | S | 0.869 | 0.160 | 5.434 | < 0.001 |
| *Phacelia ciliata* | NS | 0.522 | 0.160 | 3.263 | 0.001 |
|  | SS | 1.270 | 0.173 | 7.329 | < 0.001 |
|  | S | 1.008 | 0.160 | 6.304 | < 0.001 |
